# Structural and Logical Analysis of a Comprehensive Hedgehog Signaling Pathway to Identify Alternative Drug Targets for Glioma, Colon and Pancreatic Cancer

**DOI:** 10.1371/journal.pone.0069132

**Published:** 2013-07-23

**Authors:** Saikat Chowdhury, Rachana N. Pradhan, Ram Rup Sarkar

**Affiliations:** Chemical Engineering and Process Development, CSIR-National Chemical Laboratory, Pune, Maharashtra, India; University of Nebraska Medical Center, United States of America

## Abstract

Hedgehog is an evolutionarily conserved developmental pathway, widely implicated in controlling various cellular responses such as cellular proliferation and stem cell renewal in human and other organisms, through external stimuli. Aberrant activation of this pathway in human adult stem cell line may cause different types of cancers. Hence, targeting this pathway in cancer therapy has become indispensable, but the non availability of detailed molecular interactions, complex regulations by extra- and intra-cellular proteins and cross talks with other pathways pose a serious challenge to get a coherent understanding of this signaling pathway for making therapeutic strategy. This motivated us to perform a computational study of the pathway and to identify probable drug targets. In this work, from available databases and literature, we reconstructed a complete hedgehog pathway which reports the largest number of molecules and interactions to date. Using recently developed computational techniques, we further performed structural and logical analysis of this pathway. In structural analysis, the connectivity and centrality parameters were calculated to identify the important proteins from the network. To capture the regulations of the molecules, we developed a master Boolean model of all the interactions between the proteins and created different cancer scenarios, such as Glioma, Colon and Pancreatic. We performed perturbation analysis on these cancer conditions to identify the important and minimal combinations of proteins that can be used as drug targets. From our study we observed the under expressions of various oncoproteins in Hedgehog pathway while perturbing at a time the combinations of the proteins GLI1, GLI2 and SMO in Glioma; SMO, HFU, ULK3 and RAS in Colon cancer; SMO, HFU, ULK3, RAS and ERK12 in Pancreatic cancer. This reconstructed Hedgehog signaling pathway and the computational analysis for identifying new combinatory drug targets will be useful for future *in-vitro* and *in-vivo* analysis to control different cancers.

## Introduction

Signal transduction system represents an elegant circuitry of the cell that translates external and internal cues into appropriate cellular responses. These signaling pathways are generally organized into three main parts: Input, Intermediate and Output [1], which comprise of several proteins that mediate, signal reception, transduction, amplification and response generation. Recent advances in molecular and computational approaches have shown that a signal upon interaction with a receptor generates an intricate excitation pattern rather than a “molecular one-way path” and certain malfunction of this pattern can cause serious pathological diseases such as cancer, tumorigenesis etc. in the organisms including human. It is also a well known fact that few diseases are nothing but perturbations in signaling cascades that manifest a molecular level interaction into phenotypic changes. For example, cancer is one such “systems biology disease”, which convert a singular perturbation into a widespread excitation pattern [2]. These perturbations are not restricted to a particular cell but also affect surrounding tissues. In order to design new therapeutic strategies for such diseases, it therefore appears to be essential to investigate networks of pathways and systems at different levels of complexity rather than looking into an individual bio-molecule or chemical component. Hence, there is a need for a comprehensive study of signaling pathways for exploring these pathological manifestations, its relation with various diseases and to identify a single or combination of individual molecules that govern several different system behaviors or malfunctions.

Several concerted efforts are being made to dissect different signaling pathways, such as MAPK, Apoptosis, mTOR etc. and the related molecular mechanisms that control the cancer development of a cell or tissue in an organism [2]. Among different signaling pathways, Hedgehog is of great biological relevance as it is strongly implicated in cancer development [3]–[5]. Hedgehog is an evolutionarily conserved developmental pathway that is widely implicated in controlling various cellular responses. This pathway has a cardinal role in different cellular processes such as embryogenesis, maintenance and repairing of tissue, and homeostasis. Hedgehog signaling pathway also controls developmental processes by the interaction of Hedgehog ligands, Sonic Hedgehog (SHH), Desert Hedgehog (DHH) and Indian Hedgehog (IHH) with Patched receptors (PTCH1/PTCH2), leading to the release of Smoothened (SMO) from Patched-induced suppression [6]. SMO activation further activates the downstream components like STK36, SUFU which inhibit assembly of GLI degradation complex and thereby stabilizing GLI proteins that ultimately activate Hedgehog target genes, such as CYCLIN D2, FOXM1, SFRP, JAG2 etc. [6]. Controlled regulation of this pathway activates these target genes at certain level and thereby maintains the proper development of cell or tissue. But deregulation of this pathway can cause up or down regulation of these target genes and may cause severe outcomes in tissue or organ development. Since, this pathway is also strongly implicated in cell-renewal in adult tissues; system-component malfunctioning of this pathway can mostly lead to cancer in various cell lines of human [7], [8]. Moreover, the role of few important proteins has been identified in this pathway, such as PTCH1, SMO, GLI etc., which are mainly responsible for the malfunctioning of this pathway in various types of cancers [9]–[12]. Follow-up studies by several research groups have developed therapeutic strategies to inhibit the actions of these proteins in various cancers, but none of them achieved complete success to cure a particular cancer that is caused by abnormal activation of the Hedgehog pathway [13]–[15].

The flow of molecular excitation in any signal transduction pathway follows a complex branching pattern of cascade, therefore it is worth mentioning that targeting an individual protein in signaling pathways, such as Hedgehog, would not be fruitful to prevent its malfunction in a cancer situation. A current review by Li et al. [15], underscores the importance of combinatorial drug targets to shut off Hedgehog signaling for cancer treatment. For example, it is known that activation of cytoplasmic GLI (zinc finger transcription factor) which initiate the activity of this pathway could be regulated in two ways: *(i)* the ligand dependent way in which extracellular response i.e. hedgehog ligands interact with receptor proteins PTCH1/PTCH2 and activates G-coupled protein SMO, and *(ii)* the malfunction of the other proteins that are present in the cytoplasm which inhibit or activate its activity in the absence of hedgehog ligands. Unfortunately, till now most of the studies have mainly focused to develop a drug that will only inhibit the GLI activation, caused by the ligand dependent way. In this case, most of the study is only directed to identify the drug molecule that could suppress either PTCH1 or SMO in the membrane [16]–[19]. These drugs, such as Cyclopamine, Vismodegib etc. are only effective when a cancer cell with excessive Hedgehog pathway activation, is encountering over-expressed hedgehog ligands (SHH, IHH or DHH) or has mutated PTCH1 or SMO in membrane. Therefore, it is clear that administration of the above mentioned drugs may not be able to cure the cancers caused by some other intracellular proteins apart from sole mutation in PTCH1 and SMO. In order to overcome this problem, identification of alternative targets or a combination of drugs may be useful for successful cancer therapy.

Identification of drug targets by experimental approach sometimes becomes difficult as it requires more time and resources. Moreover, the complex regulatory networks of gene expression, entire networks of the metabolic reactions and large-scale proteomics data are now available to study response of pathways (modules) to different perturbations. Given the vast amounts of data at each level, it is a challenge to interpret the information emanating from individual assays and integrate results from multiple levels. Recent developments in integrative approaches, bioinformatics tools, mathematical and computational methods have become indispensable in understanding and analyzing such data from experimental studies. Diverse approaches for qualitative and quantitative methods and modeling of signaling pathways have been used to answer several biological questions in signaling systems [20]. The types of approaches used primarily depend on the availability of pre-existing data and type of biological questions to be answered [20], [21]. Unfortunately there are very few computational studies on Hedgehog signaling pathway [22]–[25]. All these models explore very specific themes and do not include the diseased conditions, specifically Glioma, Colon and Pancreatic cancers, which may be caused due to malfunction in Hedgehog pathway. Therefore it is necessary to reconstruct a comprehensive map of Hedgehog pathway and to study the detail molecular interactions in both normal and cancer conditions through qualitative analysis.

Moreover, identification of a combination of proteins as a drug target in Hedgehog pathway for cancer therapy requires the complete understanding of the entire mechanisms of this pathway in human cell. In order to achieve this, one needs comprehensive and most up to date information or a map of Hedgehog pathway that may help to analyze the pathway more deeply and accurately. Unfortunately, as far as the literature and biological signaling database are concerned, there is no comprehensive pathway map available for studying the Hedgehog pathway. Even, search of different popular database (See Table S1 of Text S1) revealed that there are some variations in the number of molecules and interactions reported for this pathway (See Table S2 of Text S1). This heterogeneity between database information creates immense problem for collating information to construct a comprehensive map. In some cases even there is missing information about different molecules or interactions, which are already available in experimental studies but not updated in the database. These pose a challenging problem for the researchers to get a general structure of this signaling network.

In this paper, collating the data from different database and literature, we present a master model of the Hedgehog pathway. Our extensive data mining and text extraction procedures from literature sources helped us to identify many proteins and their interactions that were not included in the existing database. To the best of our knowledge in this article we have presented a Hedgehog pathway map that is the largest Hedgehog pathway map of human till date. In comparison to existing popular database, the newly reconstructed Hedgehog map consists 57 proteins, 6 cellular or phenotypic expression and 96 hyper-interactions, which is highest. In Figure S1, a Venn diagram was constructed to compare between the number of proteins available in major database models and the proteins considered in our model. It is clear from this diagram that most of the proteins included in our model (represented by non-overlapping region), are not fully available in any of the mentioned databases except the proteins from KEGG, PATHWAY CENTRAL, BIOCARTA and PROTEIN LOUNGE. But only a subset of proteins specific to Hedgehog signaling pathway from NETPATH and GENEGO is included in our model and the rest are taken from literature and other database. Using this pathway map we then performed structural analysis using graph theoretical approach and logical analysis using Boolean formalism to understand the structure and topology of the whole network as well as to identify important proteins. We also showed that a Boolean representation of the interactions of the pathway provides an overall understanding of the system behavior by validating the model with experimental data and performed a systematic perturbation analysis to identify key drug targets for three types of cancers, such as Glioma, Colon and Pancreatic. Our main objective was to identify probable drug targets *in-silico* that could be used for future *in-vitro* or *in-vivo* analysis. From our model and computational study of the Hedgehog signaling pathway, we identified few novel combinations of proteins that could be used as a drug targets for cancer therapy.

## Results

### Reconstructed Hedgehog Signaling Pathway (Human Cell Specific)

**Figure 1:**
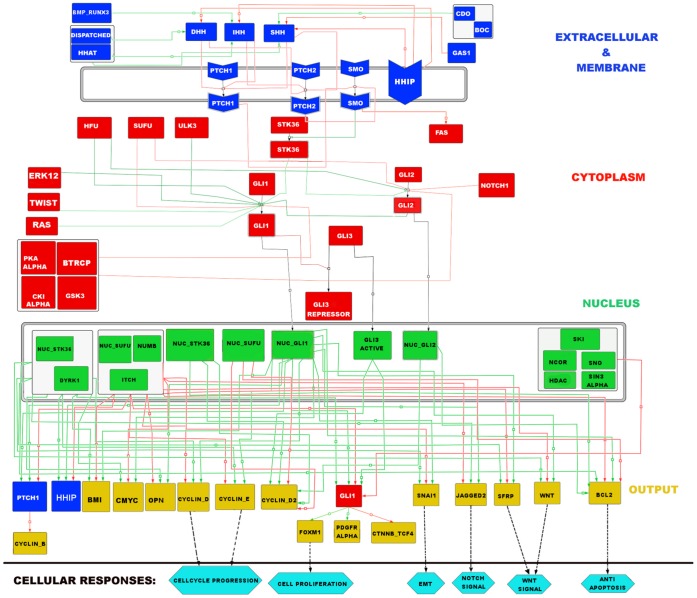
Reconstructed Hedgehog Pathway. Total 57 proteins included in this pathway figure. The green and red arrows are indicating Activation/Production and Inhibition process respectively. The black arrows indicate the nuclear translocation process. All the proteins of this network are allocated into four main regions with different color codes: Extracellular and Membrane (Blue); Cytoplasm (Red); Nucleus (Green); and Output (Yellow). The output proteins are linked with various cellular responses (cross talk with other pathways or phenotypic expressions) with black dotted arrow.

In this work, one of our main objectives was to provide an extensive and up to date Hedgehog signaling map that can serve both experimental as well as theoretical biology communities. In Figure 1, we presented a newly reconstructed Hedgehog pathway map, which to the best of our knowledge is the largest map of Hedgehog pathway till date. There were total 57 proteins (52 core proteins and 5 cross talk protein molecules from other pathways) and 96 hyper-edges included manually in the pathway figure by using the information from different sources (see the Methods section and Table S1 & S2 of Text S1).

In Figure 1, the green and red arrows signify the activation/production and inhibition events respectively. The black arrows are indicating the nuclear translocation of activated GLI transcription factors into the nucleus. In order to understand and distinguish the hedgehog component proteins according to their cellular locations, we allocated all the proteins according to four main regions: Extracellular & Membrane, Cytoplasmic, Nuclear and Output/Produced with four different colors: Blue, Red, Green and Yellow, respectively. The cross talks and phenotypic expressions of this pathway were named as “Cellular Responses” and were connected with output/produced proteins by dotted black arrow. The following are the descriptions of the proteins of each region in our reconstructed Hedgehog signaling network.

#### Extracellular and membrane

In this region, we included three hedgehog ligands: Sonic Hedgehog (SHH), Indian Hedgehog (IHH) and Desert Hedgehog (DHH). These are the ligands that bind to the receptor proteins Patched1 (PTCH1) and Patched2 (PTCH2) of a hedgehog target or responsive cell [26], [27]. Previous studies have proven that in the absence of any of these hedgehog ligands, PTCH1/PTCH2 inhibit another trans-membrane G-coupled protein “Smoothened (SMO)” within the cell membrane [26], [27]. It has been studied that this inhibition is withdrawn after the HH ligands bind to the Patched receptors. As a result of this ligand-receptor interaction SMO gets active and subsequently activate the Serine/Threonine kinase 36 (STK36) in its downstream cytoplasmic region of cell. This STK36 kinase protein is one of the major potential activators of Glioma-associated protein (GLI) in cytoplasm [6] and is called “Ligand dependent GLI activation”. In this region, the membrane proteins have been shown as a special hexagonal structure used in CellDesigner graphical notations [28], [29]. There were total 3 ligands, 6 extracellular proteins and 4 membrane proteins included in Extracellular and Membrane region.

#### Cytoplasmic proteins

In this region, we included total 16 protein molecules. All the three isoforms of GLI transcription factors GLI1, GLI2 and GLI3 were included. GLI was found in Cytoplasm as well as in Nucleus and was the main target component protein for Hedgehog pathway activation [30]. Also, there were other proteins in this region that directly or indirectly influence the three isoforms of GLI protein in the cytoplasm. These proteins were Human Fused (HFU), Unc-51-like kinase 3(ULK3), ERK1/2, RAS and TWIST [31]–[34]. It should be mentioned that ERK12, RAS, TWIST, FAS and NOTCH1 are not the hedgehog pathway proteins, although we considered these proteins as they had significant direct interactions with core proteins GLI1, GLI2 and SMO. Also their role in the ligand independent hedgehog pathway activation in Glioma, Colon and Pancreatic cancer scenarios was also an important factor for considering them in our newly reconstructed Hedgehog model. It was found that mutation or over expression of these proteins can activate GLI in cytoplasm without the help of any Hedgehog ligands. On the other hand, from various literature, we also found some repressors of GLI proteins in the cytoplasm, like Protein Kinase A (PKA), Beta-transducin repeat-containing protein (BTRCP), Casein kinase isoform alpha (CKIα), Glycogen synthase kinase-3 (GSK3) [35], [36] and included them in the network.

#### Nuclear proteins

In the nuclear region of the Hedgehog pathway map, we included 13 molecules those were mainly transcription factor, co-activator or co-repressor. The activated transcription factors GLI1, GLI2 and GLI3 translocate into the nucleus as Nuclear GLI1 (NUC_GLI1), Nuclear GLI2 (NUC_GLI2) and GLI3 active (GLI3_A) [37] respectively and help to transcribe various hedgehog target genes with the help of transcription co-activators Nuclear STK36 (NUC_STK36) and Dual specificity tyrosine-phosphorylation-regulated kinase 1 (DYRK1) proteins [38]. Also, there were few transcription co-repressors in the nucleus which were found from various literature sources and they down regulate the GLI transcription factors. These proteins, Nuclear SUFU (NUC_SUFU), NUMB, ITCH, SKI, Nuclear Receptor Co-repressor (NCOR), SNO, HDAC and SIN3A [39], [40], were included in the network. In nucleus NUC_GLI1 transcription factor transcribes the genes *ptch1, hip1, gli1* along with several other responsive genes of this pathway. In order to reduce the complexity in the pathway figure, we did not include any gene or m-RNA in this nuclear region.

#### Output proteins

This region does not specify any cellular location. We included this section separately to identify the proteins produced at the end of Hedgehog pathway. We considered a signaling network as an input-output system where the ligands and extracellular proteins were the inputs, the proteins produced as a response to these inputs at the end of this pathway could be thought as Output proteins. There were total 15 proteins including GLI1, PTCH1 and HHIP included in this section. The total numbers of proteins shown in this region are highest compare to any other published human specific Hedgehog pathway map to the best of our knowledge. Besides, all the proteins in this region were colored as yellow, except PTCH1, HHIP and GLI1. The reason was, after the production or translation, these three proteins translocate to their corresponding cellular locations and perform their activity in the pathway. In order to show this feedback mechanism, we kept their color similar to the color coded in their actual cellular location. Production of PTCH1 and HHIP proteins in this pathway switch “ON” a “negative feedback” mechanism and thus control further hedgehog pathway activation through ligand dependent way. It was experimentally proved that the Hedgehog Interacting Protein 1(HHIP) represses the Hedgehog ligands by directly binding with them and the higher concentration of PTCH1 in membrane would help to repress further SMO activation [41]–[43]. On the other hand, production of GLI1 helps to activate the pathway again and thus creates a “Positive feedback” loop in this network.

#### Cellular responses

In order to show the cross connections of the output proteins with the other pathway or cellular functions, we kept this section at the end of our pathway figure. There were 6 cellular responses included which were Cell Proliferation, Cell cycle progression, Anti-Apoptosis, Epithelial–Mesenchymal Transition (EMT), Wnt signal and Notch signal. We showed the connections of produced proteins with these cellular responses by black dotted arrow in the pathway figure.

To understand the detail activity of these molecules in the Hedgehog pathway and to perform the structural analysis, we modeled the reconstructed pathway map by two approaches: Graph theoretic and Logical (see the Methods section).

### Structural Analysis

**Figure 2:**
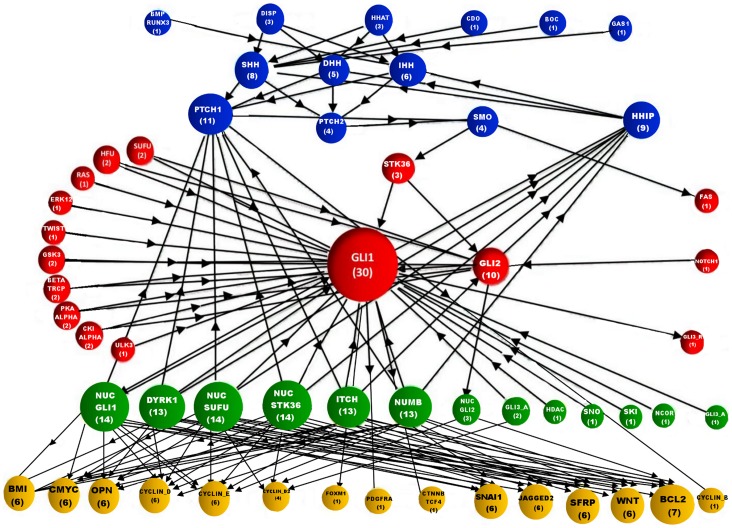
Network picture of Hedgehog signaling pathway. The colored circles represent the nodes or proteins of the pathway and the black arrows indicate an edge or connection between two nodes of the network. The nodes are colored according to their sub cellular locations in the cell (Figure 1) and are divided into four regions: Extracellular and Membrane (Blue), Cytoplasm (Red), Nuclear (Green) and Output proteins (Yellow) respectively. The size of the nodes is assigned according to their total number of connections or degree. Total degree of each node is followed by the name of the proteins. The “Bow-Tie” structure of the Hedgehog signaling network is easily visible, where the signals are converging towards GLI1 or GLI2 and diverging to its subsequent steps. The size the node GLI1 is biggest as it has highest number of connections or degree among all other proteins in the network.

The reconstructed Hedgehog pathway (Figure 1) helped us to find out the structure and topological features of this network. We used ‘Graph theory’ for this purpose. This kind of analysis is also useful for visual and/or topological interpretation of a very large complex network [44]. In our study, we considered the whole signalling pathway as a network where the signal from the hedgehog ligands traverses from extracellular region to the nucleus of a target cell via various cytoplasmic intermediate proteins. Our hedgehog signaling network was like a ‘Bow-Tie’ network and consist of 57 nodes or proteins (52 core and 5 non-core proteins of hedgehog pathway) and their 140 directed edges (interactions, regulations or the direction of flow of signal). As we know that the flow of signal of an intracellular signaling network maintains a particular direction, so we considered our graph theoretic model as a ‘Directed Graph’ or ‘Digraph’. In order to show only the connections of the proteins within the Hedgehog map, we did not include the Cellular responses in the graph theoretical model. The whole network picture is shown in Figure 2.

In Figure 2, the colored circles represent the nodes or the proteins of the network and the black arrows indicate the directions of connections or edges between two nodes. The nodes of this network were colored according to their sub-cellular location as described in Figure 1. The output proteins GLI1, PTCH1 and HHIP were not shown in the “output” region but were presented as reverse connections from NUC_GLI1 to the GLI1 of cytoplasm and to PTCH1 and HHIP of membrane region. The size of the nodes in this network (Figure 2) was assigned according to their total number of connections or degree value. GLI1 in cytoplasm had highest number of total degree in the network; therefore the size of this node in the network was largest among all the other nodes. It was also clear from this figure that the hedgehog signals from the inputs (extracellular and membrane proteins) converged to the particular proteins (GLI1 and GLI2) in cytoplasm to activate it and after its activation these proteins send the signals (actually translocate into the nucleus) to activate the production of the various target genes/proteins (like OPN, BCL2, GLI1, HHIP etc.) at the downstream of hedgehog pathway. Therefore, we can say that the flow of hedgehog signal from extracellular-membrane region to the downstream target proteins of hedgehog pathway mainly depends on the intermediate cytoplasmic GLI proteins. Due to this reason the canonical hedgehog pathway is also called as ‘GLI mediated hedgehog pathway’ [45].

We further analyzed this network from three perspectives: i) Connectivity ii) Centrality and iii) All pairs shortest path.

#### Connectivity analysis

**Figure 3:**
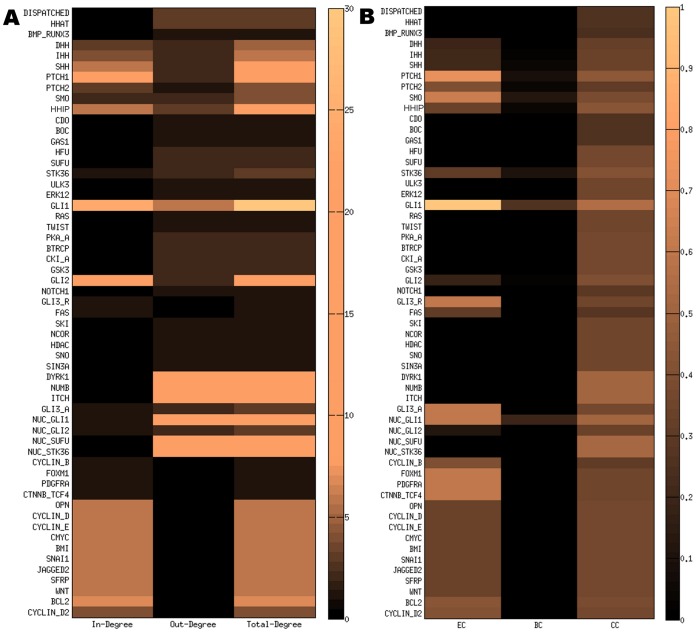
Parameter values from Connectivity and Centrality Analysis. Heat map of the values of the parameters used in Connectivity and Centrality analysis. The names of the proteins or nodes are arranged row wise (Y-axis) according to the position of their corresponding region (Figure 1). The parameter values are arranged column wise (X-axis) in the heat map. (A) Heat map of the values of the parameters values used in connectivity analysis: IN-DEGREE, OUT-DEGREE and TOTAL DEGREE of each protein. High IN-DEGREE value of GLI1, PTCH1, HHIP and SHH indicates their higher number of up-regulation by the other proteins in the network. High OUT-DEGREE value of several nuclear proteins (e.g. DYRK1, NUMB, NUC_GLI1, NUC_SUFU, NUC_STK36 etc.) refers their ability to regulate other proteins in HH network. In case of total degree, GLI1, GLI2 and NUC_GLI1 have significant highest value. It refers that these two proteins are mostly connected to the other proteins in the network. (B) Heat map of the individual centrality score of each protein of Hedgehog map. The Centrality measurement parameters used in this analysis were Eigenvector (EC), Betweenness (BC) and Closeness (CC) centrality. It is observed that GLI1 has the highest value for each parameter score. Subsequently, PTCH1, PTCH2, HHIP, STK36, NUC_GLI1, NUC_GLI2 etc. are also showing significant value for each individual centrality score.

We performed this analysis to know the number of connections of each protein with all other proteins in the network. Three types of parameters (IN DEGREE, OUT DEGREE and TOTAL DEGREE) were used in this section (see the Methods section). We calculated and presented these three parameters for each protein of the Hedgehog signaling network in Figure 3A.

The heat map (Figure 3A), representing the values of the parameters (IN-DEGREE, OUT-DEGREE and TOTAL DEGREE), shows the proteins row wise according to their cellular locations in a cell (top to bottom) and the parameter values column wise. The average IN and OUT DEGREE (all together) of the network was calculated as 2.45 and the average total degree was 4.91. In order to identify the important proteins from this heat plot on the basis of the connectivity parameters, we extracted the proteins which had the parameter values higher than their corresponding average values. All the extracted significant proteins on the basis of this hypothesis were listed in Table 1. We found that there were total 19, 10 and 23 proteins which had the higher values than the average IN-DEGREE, OUT-DEGREE and TOTAL-DEGREE, respectively.

**Table 1:** Significant proteins extracted from Connectivity analysis.

Parameters	Extracellular and Ligands	Cytoplasm	Nucleus	Output proteins
**In-Degree (>2.45)**	DHH(3), IHH(4), SHH(6), PTCH1(9), PTCH2(3), HHIP(6)	GLI1(24), GLI2(8)	NOT FOUND	**[**OPN, CYCLIN_D, CYCLIN_E, CMYC, BMI,SNAI1, JAGGED2, SFRP,WNT**]**(6) BCL2(7), CYCLIN_D2[4]
**Out-Degree (>2.45)**	DISPATCHED(3), HHAT(3), HHIP (3)	GLI1(6)	**[**DYRK1, NUMB, ITCH, NUC_GLI1**]** (13), NUC_STK36 (14), NUC_SUFU (14)	NOT FOUND
**Total-Degree (>4.91)**	DHH(5), IHH(6), SHH(8), PTCH1(11), HHIP(9)	GLI1(30), GLI2(10)	**[**DYRK1, NUMB, ITCH**]**(13), **[**NUC_GLI1, NUC_SUFU, NUC_STK36**]**(14)	**[**OPN, CYCLIN_D, CYCLIN_E, CMYC, BMI, SNAI1, JAGGED2, SFRP, WNT**]**(6), BCL2(7)

From Table 1, it was clear that receptor protein PTCH1 and two transcription factors GLI1 & GLI2 had higher IN-DEGREE values compared to the other proteins in the entire network, may be due to their high regulation or interaction with other upstream proteins in the hedgehog signaling network. PTCH1 was showing higher IN-DEGREE because most of the extracellular signals pass through this receptor protein to trigger the activation of SMO protein in membrane. On the other hand the cytoplasmic GLI1 and GLI2 had high IN-DEGREE value as these proteins are the most important proteins in the network to activate the pathway. Also, among the three hedgehog ligands, Sonic hedgehog (SHH) had the highest IN-DEGREE value as its interaction with PTCH1 and PTCH2 receptors was highly dependent on the proteins DISPATCHED, HHAT, CDO, BOC and GAS1 at the extracellular region of hedgehog target cell. The proteins in the nucleus like NUC_GLI1, NUC_GLI2, DYRK1 etc. had highest out-degree value compared to the other proteins in the network. Mainly the output proteins were connected to the outgoing connections or edges of these nuclear proteins in the network structure. Due to the presence of the higher number of outgoing connections from the nuclear proteins to the output proteins, the OUT-DEGREE values of these proteins were increased in comparison to the other proteins in the whole network. We also observed that except the nuclear proteins, the proteins from the other sub cellular locations or regions did not show significant OUT-DEGREE values.

We also extracted the proteins which had the TOTAL-DEGREE higher than the average total-degree 4.91. Table 1 shows that in extracellular and ligands region PTCH1, HHIP, SHH, IHH had significant number (greater than the average total degree) of connections or total degree in the network. It means, in order to transmit the hedgehog signal from extracellular to the intracellular region of a cell, these proteins play most effective role within the whole network. It was clear from Figure 2, that GLI1 had the highest TOTAL-DEGREE value among all other proteins in the Hedgehog signaling network. It signified that this was the most important protein in the hedgehog signaling network. Out of 57 proteins in the network, it was connected to 30 proteins. Therefore in terms of signaling network, it was the biggest ‘Hub’ in the entire network, which was actually influenced by more number of other proteins as well as influencing more proteins than the other hubs in the entire network. Similarly, in nuclear region NUC_GLI1, NUC_SUFU and NUC_STK36 formed the other larger hubs in the nucleus and thus controlling the production of various target proteins of Hedgehog pathway.

#### Centrality measurements

We measured the ‘Centrality score’ of each node or protein in the network after identifying the important “Hub proteins” from the network. In the connectivity analysis, we found some important nodes or proteins which were forming important ‘Hub’ in the whole network structure. In that case we gave the highest importance to a node on the basis of its total number of connections or degree value. Albeit in biological as well as any real world network the importance of a node or a protein does not depend only on its number of connections or neighbors [46], [47]. Sometimes the importance or significance of a node may increase due to its connections with the other important nodes in the network, though it may have lower number of neighbors or connections or *vice-versa*. “Centrality Values (*Eigenvector, Closeness and Betweenness*)”, the most useful parameter, were used to determine the relative importance of a node within a network (See the Methods section).

In this analysis, at first, we calculated ‘*Eigenvector Centrality*’ to identify the proteins according to their importance in our newly reconstructed hedgehog signaling network [46], [47]. The principle behind this parameter was that a node would be considered as an important node if it was connected to the other important nodes in the network. We calculated and presented the eigenvector centrality score for each protein in the network in Figure 3B (i.e. first column of the heat plot matrix). We presented the values of this parameter for each protein in the network and observed that GLI1and PTCH1 had high Eigenvector centrality score, but GLI2 had very poor score though it had large number of connections or degree in the network. The reason of showing this interesting feature was that in our hedgehog network (Figure 2), GLI2 was connected to NUC_GLI2, FAS, HFU, SUFU, PKA_A, BTRCP etc. that had lowest number of connections in the network. On the other hand, GLI1 was connected with another important node or protein NUC_GLI1 in the network which was regulating the expression of most of the output proteins in the network and comparably had higher number of connections than NUC_GLI2. It signified that GLI1 was connected to another most important protein NUC_GLI1 in the network. Due to this reason, the importance of GLI1 was increased significantly as compare to GLI2 protein. It was also interesting to observe that SMO, GLI3_Repressor (GLI3_R), GLI3_Active (GLI3_A) belonged to the highest important node after GLI1 and PTCH1, though they had lower number of connections or neighbors within the network.

Also, we calculated *Betweenness Centrality* and *Closeness Centrality* scores for each protein and presented these parameters in the second and third column respectively in the heat map matrix of Figure 3B [47]. We identified some important proteins of the network on the basis of these parameter values. As expected, we observed that GLI1 had the highest Betweenness and Closeness centrality score among all the other proteins in the hedgehog signaling network. Both the centrality scores of GLI1 was high because large numbers of shortest paths between two nodes were passing through it and it was connected to all other proteins with the minimum number of connections in the network. We also found that besides GLI1, there were some other proteins like NUC_GLI1, SMO, STK36 and PTCH1 had high Betweenness centrality score. On the other hand NUC_SUFU, NUC_STK36, DYRK1, NUMB and ITCH showed high Closeness centrality score after GLI1. Interestingly, all these proteins were found in the nucleus. As expected, the large numbers of the proteins that belonged to the extracellular and ligands region had lowest closeness scores i.e. they were situated more distantly from all the other nodes in the network and regulating the downstream proteins of hedgehog signaling network distantly.

#### Linear shortest paths

This parameter was used to calculate the shortest paths between every pair of nodes or proteins in the hedgehog pathway [48]. The matrix shown in Figure S2 is representing a matrix having the proteins in row and column wise and each cell represents the value of the shortest path between two nodes or proteins in the network. We found that it followed a specific pattern and observed that the shortest paths from the proteins of extracellular and ligand region to the output proteins had higher number of shortest paths (either 6 or 7), whereas the shortest paths between the ligands to cytoplasmic proteins or the cytoplasmic proteins to the nuclear proteins had lower number of shortest paths (either 2 or 3). The average shortest path of our reconstructed hedgehog signaling network was 3.581. We extracted the frequency of the shortest paths of our Hedgehog signaling network and presented the distribution in Figure S3. The frequency of the shortest path ‘3 (Three)’ in the network was highest. This observation signified that most of the proteins in the hedgehog signaling network were connected with each other on an average by 3 intermediate steps. We also found from Figure S2 that in order to transmit the signal after the bindings of PTCH1/2 proteins with ligands (SHH, DHH, IHH) to GLI1 or GLI2 proteins in the cytoplasm, it took only 3 (three) intermediate steps or links (i.e. PTCH1/PTCH2 → SMO → STK36 → GLI1/GLI2). Similarly, in order to initiate the production of the output proteins of the hedgehog pathway in the nucleus by the transcription factors GLI1, GLI2 or GLI3_ACTIVE, it required only 2 intermediate steps or links i.e.GLI1/GLI2 →NUC_GI1/NUC_GLI2 → CYCLIN_D/CMYC/BCL2. This observation also demonstrated the strong and tightly coupled signal transmission procedure from GLI1/GLI2 to the output proteins of the hedgehog pathway. Interestingly, we found that the identified important ‘hubs’ (i.e. GLI1, PTCH1, NUC_GLI1 etc.) from our connectivity analysis, were also connected by shorter number of links to each other in the network. Therefore, it was clear that most of the important or highly connected proteins in hedgehog signaling network were well connected with each other by lower number of connections or links, which also signified the robustness of the network [24].

The Graph theoretic analysis of the hedgehog signaling network helped us to find out some important proteins in the network on the basis of some important topological parameters. The parameters which we used in our network were able to identify the proteins that played crucial role to operate the hedgehog pathway normally. Several experimental studies proved that over or under expression of these identified proteins would cause abnormal activation or inhibition of hedgehog pathway and lead to uncontrolled cell proliferation or cancer stage in various types of cell lines [6], [31]–[34], [36], [41], [42]. The limitation of this analysis was that it just captured the static picture of the network and was unable to show any diseased scenario or malfunction caused by the proteins in the pathway. In order to overcome this problem we used “Logical analysis”, where all the interactions of the network were modeled into ‘Boolean or Logical equation hyper-graph’ (see Methods section).

### Logical Analysis

We simulated the logical models of the Hedgehog pathway for normal scenario as well as for three different types of cancer i.e. Glioma, Colon and Pancreatic cancer in CellNetAnalyzer [49]–[51]. Using this technique (see the Methods section), we were able to identify the proteins that were involved in the abnormal activation of hedgehog pathway in the development of these three types of cancers.

#### Perturbation analysis and model validation

**Figure 4:**
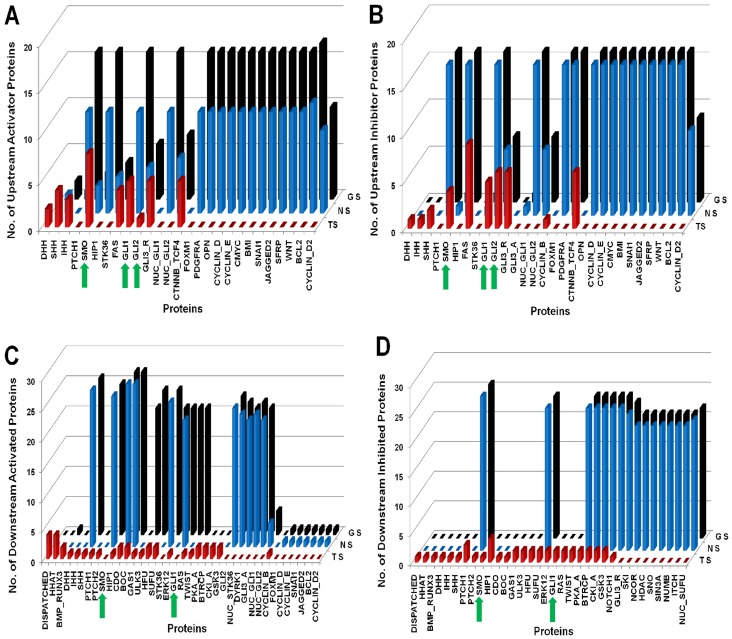
Comparison between Normal, Cancer and Perturbed scenarios for Glioma. TS: Treated Scenario; NS: Normal Scenario; GS: Glioma Scenario. The green arrow heads are indicating the minimal combination of proteins which was inhibited in the drug treated perturbation analysis. (A) Represents number of Upstream activator proteins (Y-axis) activating the proteins (X-axis) representing significant variations. Compared to the normal scenario, proteins, SHH, SMO, GLI1, GLI2, and output proteins BMI, SNAI1, BCL2 and Cyclins, are activated by maximum number of upstream activator proteins. On drug treated perturbation of SMO, GLI1 and GLI2, the number of activators of the output proteins become zero. (B) Represents number of Upstream inhibitory proteins (Y-axis) inhibiting the proteins (X-axis) representing significant variations. The numbers of upstream inhibitor proteins in normal versus Glioma scenario remain same. Similar perturbation results are observed as in (A). (C) Represents number of Downstream proteins (Y-axis) activated by the proteins (X-axis) representing significant variations. The number of downstream proteins activated in normal versus Glioma remains same. On perturbation of SMO, GLI1 and GLI2, the number of downstream proteins activated by these proteins is reduced to zero. (D) Represents number of Downstream proteins (Y-axis) inhibited by the proteins (X-axis) representing significant variations. The number of downstream proteins inhibited in normal versus Glioma remains the same.

**Figure 5:**
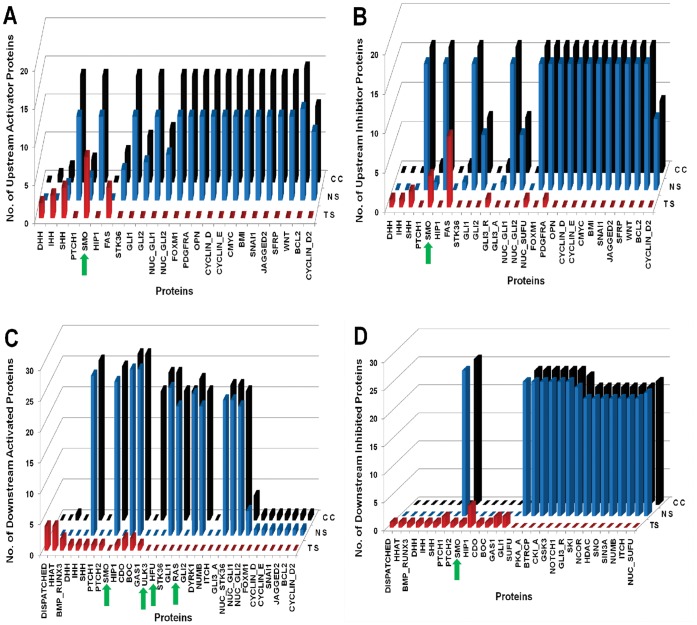
Comparison between Normal, Cancer and Perturbed scenarios for Colon Cancer. TS: Treated Scenario; NS: Normal Scenario; CC: Colon Cancer Scenario. The green arrow heads are indicating the minimal combination of proteins which was inhibited in the drug treated perturbation analysis. (A) Represents number of Upstream activator proteins (Y-axis) activating the proteins (X-axis) representing significant variations. Compared to the normal scenario, proteins, SHH, IHH, SMO, GLI1, GLI2 and output proteins BMI, SNAI1, BCL2 and Cyclins are activated by maximum number of upstream activator proteins. On drug treated perturbation of SMO, HFU, ULK3 and RAS, the number of activators of the output proteins become zero. (B) Represents number of upstream inhibitory proteins (Y-axis) inhibiting the proteins (X-axis) representing significant variations. The numbers of upstream inhibitor proteins in normal versus Colon cancer scenario remain same. Similar perturbation results are observed as in (A). (C) Represents number of downstream proteins (Y-axis) activated by the proteins (X-axis) representing significant variations. The number of downstream proteins activated in normal versus Colon cancer scenario remains same. On perturbation of SMO, HFU, ULK3 and RAS, the number of downstream proteins activated by these proteins is reduced to zero. (D) Represents number of downstream proteins (Y-axis) inhibited by the proteins (X-axis) representing significant variations. The numbers of downstream proteins inhibited in normal versus Colon cancer scenario remain same.

**Figure 6:**
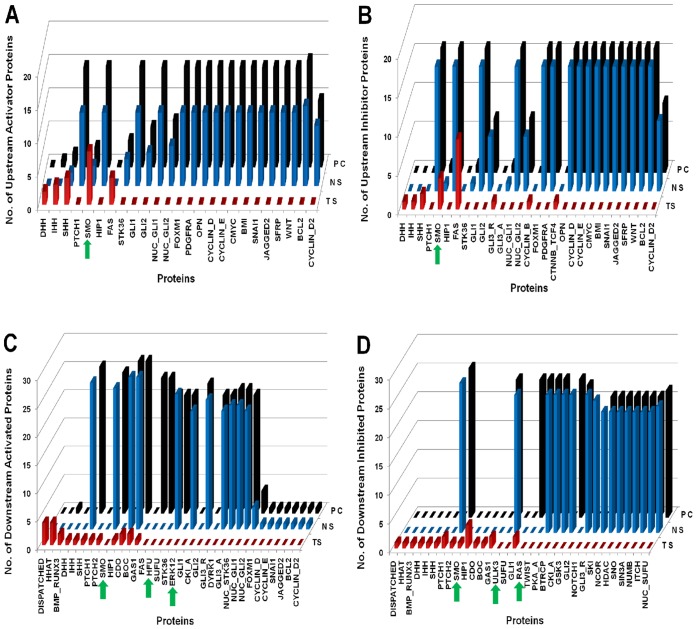
Comparison between Normal, Cancer and Perturbed scenarios for Pancreatic Cancer. TS: Treated Scenario; NS: Normal Scenario; PC: Pancreatic Cancer Scenario. The green arrow heads are indicating the minimal combination of proteins which was inhibited in the drug treated perturbation analysis. (A) Represents number of Upstream activator proteins (Y-axis) activating the proteins (X-axis) representing significant variations. Compared to the normal scenario, proteins, SHH, IHH, SMO, GLI1, GLI2 and output proteins BMI, SNAI1, BCL2 and Cyclins are activated by maximum number of upstream activator proteins. On drug treated perturbation of SMO, HFU, ULK3, RAS and ERK12, the number of activators of the output proteins become zero. (B) Represents number of upstream inhibitory proteins (Y-axis) inhibiting the proteins (X-axis) representing significant variations. The numbers of upstream inhibitor proteins in normal versus Pancreatic cancer scenario remain same. Similar perturbation results are observed as in (A). (C) Represents number of downstream proteins (Y-axis) activated by the proteins (X-axis) representing significant variations. The numbers of downstream proteins activated in normal versus Pancreatic Cancer remain same. On perturbation of SMO, HFU, ULK3, RAS and ERK12, the number of downstream proteins activated by these proteins is reduced to zero. (D) Represents number of downstream proteins (Y-axis) inhibited by the proteins (X-axis) representing significant variations. The numbers of downstream proteins inhibited in normal versus Pancreatic cancer scenario remain same.

In Figure 4, 5, and 6, we presented the normal Hedgehog scenario along with three types of cancers Glioma, Colon and Pancreatic cancer as well as their perturbed scenarios respectively. In each figure, we presented the proteins along X-axis and three types of scenarios i.e. Normal Scenario (NS); Cancer Scenario [Glioma (GS); Colon (CC); Pancreatic (PC)]; Perturbed Scenario (PS) along the Y-axis and the number of upstream activator/inhibitor proteins (A/B) or downstream activated/inhibited (C/D) proteins along the Z-axis.

In case of ‘Canonical hedgehog pathway’ (i.e. NS or Normal Scenario), we found that at time scale 2 Sonic hedgehog (SHH) was activating overall 26 different proteins (from Figure 4C, 5C and 6C) in the pathway directly or indirectly after being activated by the upstream proteins DISPATCHED, HHAT, CDO, BOC etc. Besides, we also found SMO, STK36 were activating overall 25 and 24 other protein molecules in the pathway respectively. Similar results have been shown experimentally for SHH, SMO and STK36 that are required to initiate the normal hedgehog pathway [6], [26] *i.e.* these are the main and potential activators of a normal or canonical Hedgehog pathway. Similarly we observed that in the normal scenario, cytoplasmic proteins such as GLI1 and GLI2 were activating around 21 and 23 proteins respectively. On the other hand, we found from Figure 4B–6B that the upstream inhibitors (nearly 20 protein molecules) such as PTCH2, SUFU, BTRCP, GSK3, PKA, CKI_A, NCOR, HDAC, SNO, SIN3A, NUMB and ITCH were inhibiting the activation or production of GLI1 in cytoplasm. This inhibition helps to control the over expression of GLI1 in a normal cell [35], [36], [39], [40]. The interactions between these activators and inhibitors in a canonical hedgehog pathway helped to control their abnormal activation, and consequently regulated the over production of various downstream proteins, which further result a cancerous situation in a normal and healthy cell. We observed that our model of canonical hedgehog pathway or normal scenario mimicked the expression scenarios of various proteins in a canonical hedgehog pathway of a non-malignant, non-cancerous cell line. To identify the combination of proteins as a probable drug targets, we then used this scenario as a standard model to compare against three types of cancers and subsequently perturbed few combinations of proteins for each of the cancer scenarios.

#### Glioma scenario

In case of Glioma, we considered the over expression of hedgehog ligand SHH [52], [53], i.e. the input value for SHH in our model was considered as “1” or “ON” throughout the simulation. We also considered the interactions between two kinase proteins HFU and ULK3 with the GLI transcription factors in cytoplasm and therefore considered their expression as 1 in the Boolean model. Apart from SHH, experimental evidence also showed that ERK12, TWIST and RAS proteins were up regulated during Glioma and these were known to have a direct effect on the abnormal activation of GLI1 and GLI2 [54]–[56]. Therefore to simulate the Glioma scenario in our study we considered the logical states of SHH, HFU, ULK3, ERK12, RAS, TWIST as “1” or “ON” (See Table S4 of Text S1). As several experimental studies demonstrate that over activation of GLI proteins in glial stem cell lines is the main cause of Glioma formation [52], [57], we propose that the over activation of GLI protein in Glioma happens due to the effect of the higher concentration of SHH as well as the intracellular activation or deregulation by HFU, ULK3, RAS, TWIST, ERK12 proteins in cytoplasm. The effect of the over expression of the GLI transcription factors in the cytoplasm leads to the over production of various downstream target proteins of hedgehog pathway. We also considered the down regulation or loss of function of few tumor suppressor proteins such as GAS1, SUFU, NUMB, SNO etc. (See Table S4 of Text S1). Down regulation or loss function of these proteins can also cause the up regulation of GLI proteins in cytoplasm as well as in nucleus.

In Figure 4A, we showed that the total numbers of upstream activators of SHH, STK36, GLI1, GLI2, NUC_GLI1, NUC_GLI2 and all the output proteins (OPN, BMI, SNAI1 etc.) of hedgehog pathway were higher compared to the normal hedgehog scenario. In case of Glioma, the total numbers of activators on GLI1 and GLI2 proteins were 16 and 6, whereas in case of normal scenario there were 11 and 5 proteins activating the expression of GLI1 and GLI2 proteins, respectively. These simulation results clearly showed the difference between Normal (NS) and Glioma scenario (GS). We also found the total numbers of sole activators of FOXM1 [58], PDGFRA [59], OPN [60], CYCLIN_D [61], [62], CYCLIN_E [63], BMI [64], SNAI1 [65], JAGGED2 [66], SFRP [67] were increased in Glioma scenario as compared to the canonical HH pathway. On the other hand the total numbers of upstream inhibitor proteins on these output proteins remain unchanged while comparing the Normal and Glioma scenarios (Figure 4B), the Hedgehog target cell could not nullified the over activation rate of these output proteins by their upstream activator proteins in Glioma affected cell and thus Glioma cells produced more target output proteins as compared to the normal Hedgehog pathway. Overproduction of the output proteins in a normal cell cause uncontrolled cellular proliferation and cell division [58]–[67]. On the other hand, to identify and compare the potential activators and inhibitor proteins of this pathway, we calculated the number of proteins that were activated or inhibited directly or indirectly by each proteins of the Hedgehog pathway and presented in Figure 4C and Figure 4D. We found that compared to GLI1, GLI2 was more potential activator of Glioma scenario as it was connected with GLI1 which was also an important activator in the network (See Figure 4C and 4D). Therefore it is worth mentioning that in order to suppress the GLI1 activation, GLI2 should also be suppressed. On the other hand we also found from our structural analysis that GLI1 had high eigenvector centrality compared to GLI2. This result also indicated their relative dependence in the formation of Glioma, as GLI1 was connected with NUC_GLI1, which activated most of the output proteins, whereas GLI2 was connected with NUC_GLI2 which had relatively lower number of downstream activated or inhibited molecules in the network of Glioma cell line. We also observed that HFU, ULK3, RAS, TWIST, ERK12 were functioning as sole activators on the other proteins in Glioma scenario (GS), but not in the Normal Scenario (NS). This result signify the effect of cross talk activation between core Hedgehog pathway molecules (GLI1, GLI2 etc.) with the other pathway molecules in Glioma cell line. On the other hand SHH, SMO, GLI1, GLI2, GLI3_A (core Hedgehog pathway molecules) were activating the other proteins in both normal as well as Glioma scenarios (See Figure 4C).

The above results helped us to identify the key proteins that could be used as target proteins for our perturbation analysis or combinatorial drug treatment scenario (TS). At the time of the perturbation study, we also looked into the biological relevance of that target key proteins. We identified that SHH, SMO, STK36, RAS, TWIST, ERK12, HFU, ULK3 were activating the GLI transcription factors in the cytoplasm of Glioma cell and due to this activation, the output proteins were over-expressed at the end of this pathway. It was experimentally proved that SMO plays a very crucial role to activate STK36 as well as GLI1 in cytoplasm [6]. Our graph theoretical study also proved that in ‘Extracellular and Membrane’ region SMO had significant high Betweenness centrality score (Figure 3B). Therefore, mutation of this protein in an adult glial stem cell would cause the abnormal activation of hedgehog pathway. On the other hand, blocking of this trans-membrane protein by any external drug in Glioma is helpful to reduce the activity of hedgehog signal [68]. Although SMO is important to mediate the hedgehog signal in Glioma cell as well as the activator of GLI, but still it is worth to mention that only the inhibition on SMO by external drug to Glioma cell is not sufficient to shut down the abnormal over expression of GLI proteins completely, there may be some other intracellular proteins in the cytoplasm that have the potential to activate or over express the GLI proteins without the help of SMO. Therefore we propose that there is a need to analyze the effects of this factor and development of a combinations of drugs that could suppress SMO as well as the other proteins causing the over activation of GLI.

In order to determine the other factors, we revisited our graph theoretical analysis and identified the IN-DEGREE neighbors of GLI proteins from cytoplasm. We found that the upstream activators (IN-DEGREE neighbors) of GLI1 and GLI2 proteins in cytoplasm were HFU, ULK3, ERK12, RAS and TWIST. We then selectively perturbed the logical states of these activators in Glioma scenario (GS), but unfortunately, none of the activators were able to inhibit the activation of GLI proteins alone in the cytoplasm. Only the perturbation of all these activators at a time was useful, but this combination of perturbation was not biologically feasible as several proteins had to be blocked in this perturbation study. Therefore, in order to suppress the GLI activation in cytoplasm, we directly blocked the activity of GLI1 and GLI2 in cytoplasm and also SMO in membrane of the Glioma scenario (GS) by putting ‘0’ or “OFF” as their logical states. As a result of this perturbation simulation, we observed that the expressions of the output proteins were blocked in Glioma scenario (GS).The dependency matrix obtained were used to extract the total number of proteins that were directly activated or inhibited by each protein in the pathway in this drug treated scenario (TS). The result was presented in Figure 4A–4D. In the treated scenarios (TS) (Figures 4A and 4B), it was clearly shown that the total number of upstream activator and inhibitor proteins of all the “Output proteins” of Hedgehog pathway became zero. Due to this perturbation, we also observed that the potential activators and inhibitors of hedgehog pathway (like RAS, TWIST, GSK3, BTRCP etc.) were also activating or inhibiting less number of proteins in their downstream region of the pathway (See “Treated Scenarios (TS)” of Figure 4C–4D).

#### Colon cancer

We found that in case of Colon cancer, along with IHH and SHH ligands, activated RAS/RAK pathway also up-regulated the activity of the GLI proteins in colorectal cancer cell [69], [70]. Experimental evidences have shown that over expression of SHH and IHH can cause up regulation of GLI proteins, or over activation of cytoplasmic protein RAS can also up regulate GLI proteins in Hedgehog pathway and are responsible for Colon cancer [71]–[75]. We found that in our simulation result of Colon cancer model, hedgehog ligands IHH and SHH, RAS in cytoplasm were also activating the GLI transcription factors. We also considered the down regulation or loss of function of few tumor suppressor proteins such as GAS1, SUFU, NUMB, SNO etc., while simulating the colon cancer scenario, which in normal situation inhibited the GLI proteins (See Table S4 of Text S1). As a result the activation level of GLI1, GLI2 and GLI3_A proteins by the upstream activators was seen to be greater than the normal scenario. As a result, the total number of upstream activators of the output proteins (yellow in Figure 1 and 2) of Hedgehog pathway also increased in colon cancer scenario (by comparing the normal or “NS” and colon cancer “CC” scenarios of Figure 5A). Therefore, it was clear that the abnormal activation of GLI proteins in colon cancer cell lines not only happened due to the abnormal expression of hedgehog ligands but also due to the effect of the interactions of RAS with GLI1 and GLI2. Also, HFU and ULK3 will be expressed as these are the auto-phosphorylated kinase proteins present in cytoplasm to activate GLI proteins [76], [77]. The relative potential of activating the other molecules between GLI1 and GLI2 was also measured and we found that GLI2 had higher number of downstream activated species in the colon cancer scenarios (Figure 5A–5C), as it had connection with GLI1.

These findings helped us to identify the combination of potential drug targetable proteins in Hedgehog pathway for Colon cancer cell lines. We perturbed the activation signal from SHH and IHH via PATCHED (PTCH1/2) and SMO proteins to GLI transcription factors and the interactions between HFU, ULK3 and RAS with GLI proteins. In order to do that we put the logical state of SMO, HFU, ULK3 and RAS as ‘0’ (Zero) and performed the combinatorial drug perturbation or Treatment simulation (TS). We found that the total number of the activated proteins by GLI1, GLI2 and GLI3 were decreased and the expression of the HFU, ULK3 and RAS proteins were blocked (comparing Colon cancer and Treated scenario of Figure 5C). In Figures 5A and 5B, we showed that the activation and inhibition level of GLI transcription factors in nucleus and cytoplasm by the upstream proteins were reduced; as a result, the activation of output proteins were blocked.

#### Pancreatic cancer

In case of pancreatic cancer, we found the expression of IHH, PTCH1 and SMO in pancreatic cancer cell line, which signified their role of hedgehog pathway activation in this type of cancer formation [78]. Simultaneously, over expression of cytoplasmic proteins such as RAS and ERK12 in the pancreatic cancer cell line was also reported in experimental study [33], [79]. Several pathological and biopsy reports on pancreatic cancer have been found that the mutation or over expression of these proteins are causing the cancer in Pancreas [68]. We also included the down regulation or loss of function scenario of few proteins such as GAS1, SUFU, NUMB, SNO etc. (See Table S4 of Text S1) in pancreatic cancer model. As a result, perturbation of SMO or PTCH1/PTCH2 receptors was not effective to reduce the activation of GLI1/GLI2/GLI3_A in pancreatic cancer model [80]. Therefore, in order to completely repressed the over expression of GLI proteins in cytoplasm, we also had to perturb the logical states of RAS and ERK12 in our Boolean model of pancreatic cancer scenario. We also perturbed the logical state of HFU (human homologue of Fused protein) and ULK3 as these were the common and essential auto-phosphorylated kinase proteins for enhancing the activation of GLI1 and GLI2 in cytoplasm [76], [77]. *In-silico* treatment of the pancreatic cancer model by the perturbation of these proteins caused the suppression of GLI transcription factors in cytoplasm and subsequently the inhibition of the Hedgehog target or output genes/proteins. We observed that the SMO, HFU, ULK3, RAS, ERK12 proteins were repressed and subsequently the expression of GLI transcription factors in cytoplasm were down-regulated. Now, down-regulation of GLI proteins in cytoplasm caused the down regulated productions or the transcription of various target oncogenes or oncoproteins of Hedgehog pathway like BMI, FOXM1 etc. The expression of various output proteins like OPN, BMI, SNAI1, JAGGED2, PDGFRA was not observed in our drug treated perturbation scenario (TS). In Figures 6A and 6B, we compared the total number of upstream activators and Inhibitors of each protein for Normal, Pancreatic and Treatment scenarios respectively. Whereas, Figures 6C and 6D represented the comparison of total number of downstream activated and inhibited proteins by each protein of the three scenarios respectively. In Pancreatic cancer model, we also observed that GLI2 had higher number of downstream activated proteins compared to GLI1 (Figure 6C) as it had an activation effect on GLI1, although the Eigenvector centrality of GLI2 was low compared to GLI1. Other signaling pathways like Wnt, Notch was also blocked by this perturbation analysis. Blocking the cross talk with Wnt and Notch signaling pathway would help to suppress the growth of cancer cell, as these pathways are also involved in pancreatic cancer. The entire simulation result of pancreatic cancer scenario was summarized in Figure 6A–6D.

**Figure 7:**
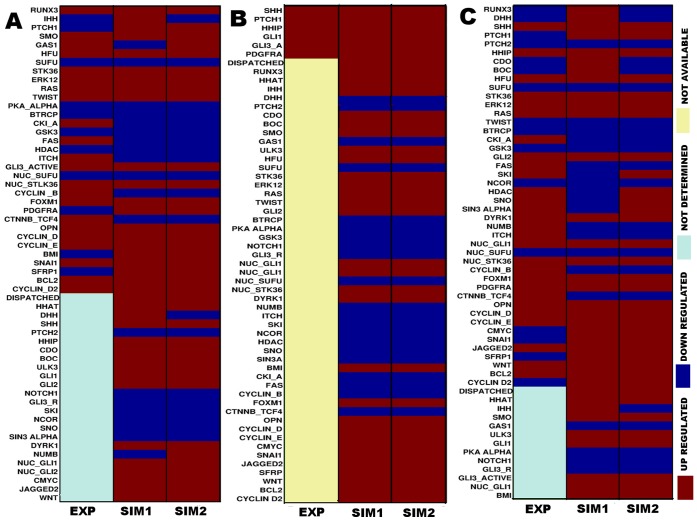
Comparison of protein expression levels observed in experiment and model simulation for different cancer situations. EXP: Experimental data observed from published literatures; SIM1: Simulation 1 performed using the expressions data from Table S4 of Text S1; SIM2: Simulation 2 performed using the expressions levels observed from experimental data. (A) Represents the comparisons of the expressions of hedgehog pathway proteins found in the experimental data (EXP) of Glioma Grade IV cell line [99] and in the corresponding simulation (SIM1 and SIM2) data from our logical model. (B) Represents the comparisons of the expressions of hedgehog pathway proteins found in the experimental data (EXP) of Colon cancer cell line [101] and in the corresponding simulation (SIM1 and SIM2) data from our logical model. (C) Represents the comparisons of the expressions of hedgehog pathway proteins found in the experimental data (EXP) of Pancreatic cancer cell line [100] and in the corresponding simulation (SIM1 and SIM2) data from our logical model.

The experimentally observed expression data of each protein for all three types of cancer was presented in Figure 7 (A–C). The model was simulated in two ways to validate with these experimental observations for three types of cancers. In Simulation 1 (SIM1), we considered the logical states mentioned in Methods section and in Table S4 of Text S1. In Simulation 2 (SIM2), we considered the expression of input proteins observed from the experimental data (EXP) for each of these cancer types. It is to be noted that in the experimental data, up regulation of IHH, RUNX3, SMO, STK36, TWIST, ERK12, RAS and down regulation of tumor suppressor onco proteins SUFU were co-occurring in Glioma grade IV cell line (See the first column of Figure 7A). Similarly, in Colon cancer cell line, up regulation of SHH, GLI1, PDGFRA were co-occurring (See the first column of Figure 7B). On the other hand up regulation of SHH, STK36, ERK12, RAS and down regulation of SUFU were co-occurring in Pancreatic cancer cell line, hence in our second simulation (Simulation 2), we considered all these co-occurrence as initial states to provide biologically realistic predictions.

#### Validation of glioma, colon and pancreatic scenarios

In case of Glioma Grade IV cell line, we found the expression level (UP as Red and DOWN as Blue) of 33 proteins out of 57 proteins (See the first column of Figure 7A). Rest of the proteins showed undetermined expression level and they were grouped into the lower portion (Light Blue). Within these 33 determined proteins, our simulation (SIM1; second column of Figure 7A) had correctly predicted the expression level of 22 proteins (66.66% accuracy). This result also signify the effect of co-occurrence of the over expression of the activator proteins HFU, RAS, TWIST of hedgehog pathway in Glioma Grade IV cell line. Further using the experimental expression data, we performed Simulation 2 (See the third column of Figure 7A) and compared the outcome with both Experiment (EXP) and Simulation 1 (SIM1) (See Table S6 of Text S1). Comparing the results of Simulation 2 (SIM2) with Experimental data (EXP) (See first and third column of Figure 7A), we found that out of 33 experimentally determined proteins, we correctly predicted the expression levels of 25 proteins (75.75% accuracy). On the other hand, while comparing the simulation result between Simulation 1 and Simulation 2 (See first and second column of Figure 7A), we found that out of 57 proteins, expression levels of 54 proteins were showing same expressions levels having accuracy 94.37% (See Table S6 of Text S1). Therefore, in both the cases our model showed promising predictions as compared to experimental data of Glioma Grade IV cell line. Hence the combination of drug targetable proteins identified from our drug treatment scenario (TS) of Glioma model could be used as probable drug targets for the treatment of Glioma Grade IV specific cell line.

In case of Colon cancer scenario, we considered the protein expression of colon cancer cell line data and the up regulation of SHH, PTCH1, HHIP, GLI1, GLI3_Active and PDGFRA were identified from this experiment (See the first column of Figure 7B). The expression levels of rest of the proteins considered as “Not available” and were grouped separately (light yellow) in the first column of Figure 7B. Within these 5 determined proteins, our simulation (SIM1)) had also correctly predicted their expressions with 100% accuracy (See the second column of Figure 7B). Using our simulation we were also able to find out the expressions levels of the other proteins, whose expression levels were not available in the experimental data, which we think, was one of the advantages of our model simulation. Further, using the experimental expression data, we performed Simulation 2 (See the third column of Figure 7B) and compared the outcome with both Experiment (EXP) and Simulation 1 (SIM1) (See Table S6 of Text S1). In both the cases we observed same expression levels of the proteins (100% accuracy), which strongly validated our *in-silico* model on Colon cancer scenario. Similarly, our model simulation for pancreatic cancer cell line also showed significant high accuracy while validating with the experimental data. We were able to extract the expressions levels of 44 out of 57 proteins of our hedgehog model in pancreatic cancer cell line from the published microarray expression data. The rest of the proteins were grouped into separately (light blue) in the first column of Figure 7C. The up regulation of HFU, ERK12, RAS were observed in the microarray expression data (See the first column of Figure 7C). Within these 44 determined proteins, our simulation (SIM1) correctly predicted the expression levels of 25 proteins with 56.80% accuracy. Comparing the expressions of the proteins of in microarray expression data (EXP) and Simulation 2 (SIM2) (See the first and third columns of Figure 7C), we found that out of 44 determined proteins of micro array expression data, Simulation 2 correctly predicted the expressions level of 32 proteins with 72.72% accuracy (See Table S6 of Text S1). On the other hand while comparing the simulation results between Simulation 1 and Simulation 2, we found that out of 57 proteins, our simulation had correctly predicted the expression levels of 47 proteins with 82.45% accuracy (See Table S6 of Text S1). Therefore, it is worth mentioning that the expressions levels considered in Table S4 of Text S1 were sufficient to simulate the pancreatic cancer cell line more close to reality.

These results clearly signify that the expression values that we considered from in Table S4 of Text S1 for all the three cancer scenarios were almost correctly considered and validated our model simulations. Further it is worthy to note that the target proteins such as RAS, ERK12, HFU, ULK3, identified from the treatment scenario, are also been over expressed in the experimental data.

#### Comparison between SMO inhibition and combinatorial drug treatment

**Figure 8:**
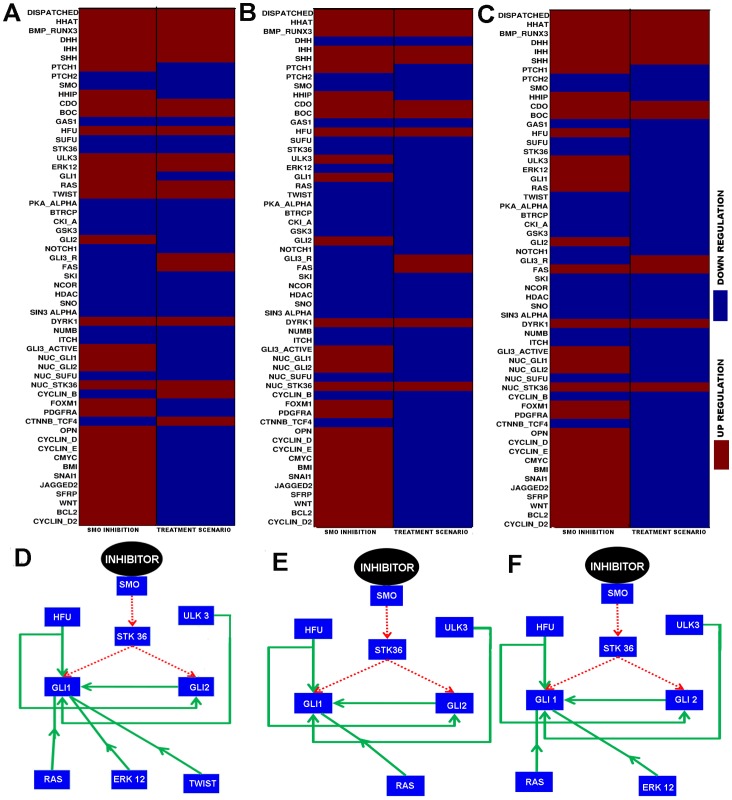
Protein expression levels observed in SMO inhibition and Treatment Scenarios for different cancers. First columns of (A), (B) and (C) represent the expressions of the proteins found after inhibiting SMO in Glioma, Colon and Pancreatic cancer models, respectively. Second columns of (A) represents the expressions of the proteins observed in the treatment scenario by perturbing SMO, GLI1 and GLI2 in combination in the same Glioma model; (B) represents the expressions of the proteins in the treatment scenario by perturbing SMO, HFU, ULK3, and RAS in combination in the same Colon cancer model; (C) represents the expressions of the proteins in the treatment scenario by perturbing SMO, HFU, ULK3, ERK12, and RAS in combination in the same Pancreatic cancer model. (D), (E) and (F) represent the identified alternative pathways (shown by solid green arrows) that remain active even after the inhibition of SMO in membrane (pathway shown by broken red arrows) by its inhibitor molecule (i.e. Cyclopamine, Vismodegib etc. ) in Glioma, Colon and Pancreatic cancer scenarios, respectively.

Earlier we mentioned (Introduction section) that the currently available SMO protein inhibitor Cyclopamine or its closed derivative drug Vismodegib were only effective in the suppression of hedgehog pathway activation in cancer cell, where the pathway was abnormally activated by mutation in SMO or by the over expressions of hedgehog ligands SHH, DHH and/or IHH. The main mechanism of these molecules was to inhibit the function of SMO, so that it could not activate GLI proteins in cytoplasm via STK36. Therefore, the effectiveness of these SMO inhibitor molecules were always questionable if the pathway got activated by some intracellular activators molecules through cross talk with other pathways or by the loss of some tumor suppressor proteins inside the cell. In this article, our main objective was to find out the effects of those cross talking molecules, such as RAS, TWIST, ERK12, NOTCH1 or the loss of functions of few tumor suppressor proteins such as SUFU, GAS1, SUFU, NUMB, SNO in the three types of cancer scenarios discussed earlier. In order to check the effectiveness of SMO inhibition in the treatment of three types of cancers we performed the model simulation by only inhibiting the SMO expression levels (*in-silico* treatment of cancer cell with SMO inhibitors) and found that SMO inhibition alone was not able to down regulate the activity of some onco-proteins such as GLI1, GLI2, GLI3_A and also the output proteins related to these. In the first columns of Figure 8 A–C, we presented these simulation results for Glioma, Colon and Pancreatic cancer cell lines respectively.

In order to identify the alternative pathways or connections present in the hedgehog pathway, which was activating the GLI transcription factors after inhibiting SMO, we calculated the dependency matrices (Figures S4, S5 and S6) for each cancer scenarios, where only the SMO activations was blocked. Using this information and from the structural analysis (i.e. the analysis of Shortest paths) of hedgehog signaling network, we were able to identify the alternative pathways which were causing the GLI activations in each type of cancer scenarios. These alternative pathways (solid green arrows) showed in Figure 8 D–F for Glioma, Colon and Pancreatic cancer cell line respectively. We observed that although the activation pathways (broken red arrows)from SMO to GLI1 and GLI2 via STK36 protein were blocked by the effect of SMO inhibitor in each cancer scenario, HFU could also activate GLI1 and GLI2 in each cancer scenario (solid green arrows). Also, in Glioma scenario, we identified that the activation pathways (solid green arrows in Figure 8D) from RAS, ERK12, ULK3 and TWIST to GLI1 could up regulate this protein, whereas the activation pathways (solid green arrows in Figure 8E) from RAS, ULK3 to GLI1 in Colon cancer scenario or the activation pathways (solid green arrows in Figure 8F) from RAS, ULK3 and ERK12 to GLI1 in pancreatic cancer scenario could also be the alternative pathways to up regulate the GLI1 protein. It is also to be noted that in each cancer scenario, there was another activation link from GLI2 which could up regulate GLI1.

Identification of these alternative pathways clearly showed that in order to suppress the hedgehog pathway activity completely in these three types of cancer cell lines, only the SMO inhibition would not be effective, as there were other molecules/proteins which were still activating the pathway. Our *in-silico* combinatorial treatment (i.e. Treatment scenarios) also accounted this constraint and as a result we proposed a need for combinatorial drug target therapy to completely shut down the hedgehog pathway activity. Using this approach, we were able to down regulate the activity of GLI proteins as well as the up regulation of the output onco-proteins (See the treatment scenarios of Figures 4, 5 and 6) by perturbing the over expressions of few optimal combinations of proteins (SMO, GLI1, GLI2 in Glioma Grade IV cell line; SMO, HFU, ULK3 and RAS in Colon cancer cell line; and SMO, HFU, ULK3, RAS and ERK12 in pancreatic cancer cell line). In order to compare the SMO inhibition with the proposed combinatorial treatment, we showed the simulation results in Figure 8 as expression matrices for these three cancer types. In the second column of Figure 8A, we showed the combinatorial inhibition of SMO, GLI1 and GLI2; and found the down regulation of various onco proteins including GLI transcription factors. We also found the up regulation of few proteins like the repressor form of GLI3 (i.e. GLI3_R), FAS and CTNNB_TCF4 complex, which were also important for inhibiting the uncontrolled cellular proliferations. Similar results were observed in the Treatment scenarios by inhibiting SMO, HFU, ULK3 and RAS in Colon cancer cell line and SMO, HFU, ULK3, RAS and ERK12 in Pancreatic cancer cell lines (See the second columns of Figure 8B and C).

## Discussion

Several attempts have been made so far to study the Hedgehog signaling pathway from experimental as well as theoretical and computational perspective. Dillon et al. [22] proposed reaction-diffusion kinetic models of Hedgehog signaling pathway to study Patched-Smoothened interaction and function of SHH as a long range morphogen. A dynamic model based approach to analyze the signal transduction and transport mechanism of Sonic Hedgehog to study tissue patterning has also been done successfully [23]. All these studies are based on either Ordinary Differential Equation (ODE) or Partial Differential Equations (PDE), the success of which immensely depends on reliable kinetic constants and initial concentrations, and hence requires substantial data availability of which is itself a much bigger challenge in reality. Since these are also computationally intensive, hence study of kinetic models for large network becomes challenging and difficult. In this context, qualitative modeling approach is much more amenable to model larger biological networks than quantitative ODE approaches. In contrast to highly specific ODE based models, logic based models can model interactions between a large number of species and can be used to train, validate and generate predictions from a model [20]. This also enables the understanding of the essence of how a system functions at a larger scale before proceeding to account for the kinetic information. Previous attempts used Boolean logic to model developmental pathways for the topological study of interactions that enable prediction of patterning in *Drosophila melanogaster* [24], and exploration of the effect of transient perturbations on development of wild type pattern for the segment polarity network [25]. But all these studies do not include the diseased conditions, specifically Glioma, Colon and Pancreatic cancers, which may be caused due to malfunction in Hedgehog pathway. Therefore, it is important to construct a comprehensive map of Hedgehog pathway and to study the detail molecular interactions in both normal and cancer conditions through qualitative analysis.

In our study, at first we reconstructed the Hedgehog signaling network using the information available from various sources and tried to include as many as possible target output proteins of this pathway. We included the connections of the output proteins JAGGED2, WNT, SFRP, CYCLIN_B, CYCLIN_D, CYCLIN_D2, CYCLIN_E, OPN, SNAI1, CMYC, BMI, BCL2, FOXM1 and PDGFRΑ with the phenotypic outcomes or cellular responses (like Cell proliferation, Cell cycle progression and Endothelial to Mesenchymal Transition etc.) and also with three other important pathways, WNT, NOTCH and Anti-Apoptosis [6], [60]–[65], [81]–[85].The inclusion of these output proteins in the reconstructed pathway map helps to understand how Hedgehog signaling pathway controls the major developmental procedures of a cell, such as cell division, cell proliferation and also the cross talks with other pathways. Also, it is important to note that most of the output proteins presented in the reconstructed map were also oncoproteins and thus correlated this pathway with various types of cancers. Also, we included ERK12, RAS, TWIST, FAS, NOTCH1, which are not the core hedgehog pathway proteins. The inclusion of these proteins in our reconstructed map was to show the regulation or cross talks of hedgehog pathway with other molecules from different signaling pathways like WNT, NOTCH, MAPK etc. Including these non-core proteins, as far as the literature and database are concerned, this reconstructed map of Hedgehog signaling pathway represents the highest number of molecules and interactions, and is considered for further computational analysis.

In this work, we performed two types of computational analysis, Graph theoretical and Boolean or Logical analysis. In order to identify the important proteins from the network, the parameters (like Degree; all pair Shortest paths; Eigenvector, Betweenness and Closeness centrality) were used in the graph theoretical analysis. We observed that GLI1 was the most important protein within the entire network and formed a “hub”, as it was showing high values in all kinds of network parameters measured in this analysis. Therefore from our analysis it can be easily assumed that perturbation (i.e. mutation, malfunction, high or low expression etc.) of this protein or node in Hedgehog signaling network will affect the normal network function and may cause several types of cancers, which is also found in experimental observation, as in Glioma, Colon and Pancreatic cancer cell lines GLI1 shows over expression [52], [72], [85]. In our study, the higher number of degree or connections showed by PTCH1 also implied that it was one of the most important proteins in the Hedgehog pathway, and this supports numerous experimental studies where mutation of PTCH1 protein was shown to affect the flow of normal Hedgehog signal and cause pancreatic and colon cancer [80], [86]. High concentration of PTCH1 protein in membrane was helpful to regulate the activated SMO protein in membrane, so that it cannot further activate GLI in cytoplasm. The high IN-DEGREE showed by SHH also implied that this ligand was mostly regulated by some other extracellular proteins at the time of binding with PTCH1/2. It has been experimentally shown that CDO and BOC both help to activate SHH ligand, whereas GAS1 inhibits it to bind with Patched proteins (PTCH1/2) in the membrane [87]. Therefore, over expression of CDO and BOC in extracellular region enhance the binding activity of SHH to bind with PTCH1/2. On the other hand higher OUT DEGREE value of nuclear proteins signified the role of these proteins to process the hedgehog input signal into the output products. Therefore, mutation or malfunction of these proteins in nucleus would cause over production of various target oncoproteins of the Hedgehog pathway. In our analysis GLI1 had shown highest IN-DEGREE, OUT-DEGREE and TOTAL DEGREE among all other proteins and thus forming the largest hub within the whole network. This result also suggested that inhibition of this protein would completely disrupt the normal flow of Hedgehog signal and thereby other cellular functions will be disturbed.

In the case of Eigenvector centrality score, GLI1, SMO, PTCH1, GLI3_A had shown significant scores. It is implied that these proteins did not have only high number of connections but also connected with other highly prestigious node that had higher number of connections in the network. It was also interesting to observe that although GLI1 and GLI2 had higher number of connections in the network, the eigenvector centrality of GLI2 was low compare to GLI1. The reason behind this kind of behavior was the connection of GLI1 with another higher central node NUC_GLI1 in the network. On the other hand while comparing the number of down-stream activated proteins of these two proteins, we found that GLI2 had higher number of down-stream activated proteins compared to GLI1. This result was also reflected in our Logical analysis, where we observed that in case of Glioma, Colon and Pancreatic cancer scenarios the number of upstream activator/inhibitor species and downstream activated species of NUC_GLI1 was high compared to the NUC_GLI2 (Figure 4, 5, and 6). Also, GLI1, NUC_GLI1, SMO, STK36 and PTCH1 had shown high Betweenness centrality score and GLI1, NUC_SUFU, NUC_STK36, DYRK1, NUMB and ITCH had shown high Closeness Centrality score in our analysis. It was clearly seen that GLI1 had significant scores in all three kinds of centrality parameters and thus we can say that GLI1 was the most centrally situated protein in our reconstructed Hedgehog signaling network. Therefore, knock out or mutation of this protein from the hedgehog signaling network would cause most significant effect in the normal cell and leads to the cancerous stage [82]. Apart from GLI1, SMO was also found as another important protein which featured high Eigenvector and Betweenness centrality scores. In order to transmit the activation signal after ligand binding to the PTCH1 and PTCH2 receptors, SMO becomes active. Due to this reason, this membrane bound receptor protein was showing high centrality scores and hence it would be the possible potential drug targetable protein in the cancer caused by Hedgehog pathway activation. The Closeness centrality score showed by most of the nuclear proteins implied that these were the proteins that were connected with lower number connections to the other proteins in the network and thus regulated by maximum number of proteins in the network. Therefore, certain disturbance in the other proteins can perturb the normal activity of these proteins in the network.

In order to analyze the importance of this signaling pathway as well as the individual proteins involved in Glioma, Colon and Pancreatic cancer cell lines, we developed Boolean or Logical models or scenarios. We found that, if we perturbed the logical states of SMO, GLI1 and GLI2 proteins from 1 to 0 in Glioma model, it will be possible to suppress the expression of various output proteins (e.g. JAGGED2, WNT, SFRP, CYCLIN_B, CYCLIN_D, CYCLIN_D2, CYCLIN_E, OPN, SNAI1, CMYC, BMI, BCL2, FOXM1, PDGFRΑ etc.) as well as the phenotypic expressions of the Glioma affected cell (Figure 4). Therefore we propose that inhibition of these proteins would be helpful for the therapeutic treatment of Glioma. We observed that there were several proteins which were activating the GLI transcription factors in cytoplasm and these proteins were connected with other signalling pathways. Therefore inhibiting those proteins could affect the normal functioning of other pathways. In order to prevent such collateral damage, we propose that, in Glioma cell line, selectively targeting SMO in cell membrane, and the nuclear translocation of activated GLI1 and GLI2 within cytoplasm would be more effective to completely shut down the Hedgehog pathway by suppressing the activity of different proteins responsible for uncontrolled cellular proliferation (Figure 4) and this was found to be the minimal combinations of proteins required. Also, inhibition of the activity of GLI2 protein was necessary to prevent its positive feedback loop to GLI1 activation.

In case of Colon cancer scenario, we found that inhibition of SMO, HFU, ULK3 and RAS was useful to suppress the expressions of various responsive proteins of Hedgehog pathway (Figure 5). Several experimental studies have already proven the correlation of the mutation of *ras* gene with the Colon cancer [88], [89]. As this protein was one of the activator of GLI1 in cytoplasm, therefore inhibition of RAS may also help to shut down the Hedgehog pathway. Hence, targeting RAS in cytoplasm would be effective to reduce the over activation of GLI proteins in cytoplasm and consequently several oncoproteins like PDGFRA, BMI, SNAI1 etc. of Hedgehog signaling. Few studies have already been reported that mutated RAS family proteins could be the suitable drug targets for treating various types of cancer [90], [91], which also supported our computational findings. On the other hand, our analysis also revealed that suppression of SMO, HFU and ULK3 by external drug would be required to completely shut down the Hedgehog signaling in the colon cancer cell line. Therefore, we propose that in order to shut down the effect of Hedgehog signaling in colon cancer cell line for *in-vitro* or *in-vivo* analysis, one could also think a combination of drugs that will suppress the activity of SMO, HFU, ULK3 and RAS proteins altogether.

From our analysis, we also suggested a minimal combination of proteins, SMO, HFU, ULK3, RAS and ERK12, the expressions of which needed to be inhibited so as to control the effect of mutated hedgehog signaling pathway in pancreatic cancer by suppressing the activity of different proteins responsible for uncontrolled cellular proliferation (Figure 6). Also, suppression of HFU and ULK3 in cytoplasm would be helpful to block the enhanced activation of GLI proteins. Experimental studies have already proven their role to activate and enhance the production of phosphorylated GLI transcription factors in the Hedgehog signaling pathway [76], [77]. This perturbation would decrease the concentration of NUC_GLI1, NUC_GLI2 and GLI3_A in nucleus and consequently bring down the production of various output proteins of Hedgehog signaling network.

The model was validated with existing microarray data for these three types of cancer (Figure 7) and also simulated using the experimental data. In both the cases it shows promising predictions. Moreover, to determine the alternative pathways which are still active under drug treatment we refined the model simulation by only inhibiting SMO in three types of cancer scenarios and compared the results with the corresponding Treatment scenarios (Figure 8). Using this analysis we found few alternative pathways in each cancer scenarios, which have the ability to up-regulate the GLI proteins without the help of hedgehog ligands (Non Canonical or ligand independent Hedgehog pathway activation).

Like all other *in-silico* models, our logical model had also some limitations, as it was unable to present a quantitative measurements of the expression levels, the rate of inhibition required in combinatorial therapy, or the dose dependent scenarios in the treatment of cancer cell lines. For that we require a dynamic model which could resolve these issues. But the model helped to identify the expression levels of different proteins which were “not determined” in the experimental data, due to lack of significant expression levels. Moreover, the proteins identified as probable drug targets from these simulations were not novel targets, and individually their efficacy as drug targets was tested experimentally. But the optimal combinations of drug targets used in these simulations were new and to the best of our knowledge, the effectiveness of targeting these proteins in combinatorial drug target therapy was not tested yet. Therefore, our *in-silico* simulations identified few “novel combinations” of drug targets and may be helpful to the experimental biologists as well as pharmacologists to experiment on combinatorial drug target therapy on Hedgehog pathway in different cancer cell lines, alternative to the ligand dependent way by inhibiting single molecule.

### Conclusion

Hedgehog signaling pathway is widely implicated in controlling various cellular responses and plays a cardinal role in different cellular processes including carcinogenesis. Malfunctioning of this pathway can mostly lead to cancer in various cell line of human. The role of few important proteins such as PTCH1, SMO, GLI etc., in this pathway has already been identified to be responsible for various types of cancers, such as Glioma, Colon and Pancreatic. Targeting this pathway in these types of cancers would be helpful, but prior to that, extensive knowledge regarding this pathway, its interactions and roles with the proteins of other important signaling pathways is necessary. Hence, there is a need for an accurate, comprehensive pathway map of Hedgehog, detailing all the species and interactions involved in the functioning of the pathway. Moreover, to control the pathway activity, most of the studies till now have mainly focused to develop a drug which only inhibit single proteins, such as GLI (ligand dependent way) or PTCH1 or SMO in the membrane and may not be able to cure the cancers caused by some other intracellular proteins apart from sole mutation in GLI or PTCH1 or SMO. Hence identification of alternative targets (single or combinatory) for administration of drugs is itself a challenging problem in this direction.

Through Reconstruction, Graph theoretical and Boolean analysis of the pathway, our study was aimed in this direction to get an insight about the signaling proteins in the Hedgehog pathway along with identification of alternate drug targets for Glioma, Colon and Pancreatic cancer, where the pathway is known to become mutated. We reconstructed a new Hedgehog signaling map by collating the data from various database and literature sources. The Hedgehog signaling map shown in this article is a large, extensive and informative network to the best of our knowledge. This map was also used for structural and logical analysis in our study. In structural analysis, we analyzed the network topology, identified few important proteins, and showed the robustness of the entire Hedgehog signaling network. The computational results are supported by available experimental observations from literature. On the other hand, in logical analysis, we constructed a single master model of the whole interactions using simple Boolean operators and created the models for normal and three types of cancer: Glioma, Colon and Pancreatic cancer. The simulations of this model also lead to identification of important proteins that can be used as probable drug targets for these cancers and the result is supported by experimental findings from literature.

Comparing the cancer scenarios with normal scenario, we found some important minimal combinations of proteins which may be used as a probable drug targets for further *in-vitro* and *in-vivo* analysis. Perturbing at a time the combinations of the cytoplasmic activated proteins GLI1, GLI2 and membrane protein SMO in Glioma scenario; SMO, HFU, ULK3 and RAS in Colon cancer scenario; SMO, HFU, ULK3, RAS and ERK12 in Pancreatic cancer scenario; we observed the under expressions of various oncoproteins in Hedgehog pathway. The effects of individual perturbations on few proteins like SMO, PTCH1, GLI1 has already been observed in the treatment of various cancers, but to achieve more accurate therapeutic strategy, the perturbation effects of these minimal combination of proteins found from our analysis have not yet been studied. In this paper, we sought for a new therapeutic strategy to inhibit the Hedgehog pathway by targeting some novel combination of proteins as a probable future drug targets. Since, our analysis was based on *in-silico* logical model and did not include any kinetic parameters; therefore we were unable to show any dynamic behaviors of the perturbation effects on the identified target proteins. But, our reconstructed Hedgehog signaling pathway and the computational method for identification of new combinatorial drug targets or pathway signatures provide a more sensible strategy for finding therapeutic targets for cancer and may be useful to the drug industry and experimental biologists to explore this pathway further. Our findings certainly pave the way for newer biomarker identification and also for therapeutic marker identification in different types of cancers.

## Methods

### Reconstruction of Hedgehog Signaling Networks

Initially we tried to construct a comprehensive map of Hedgehog pathway map from biological database. We searched 21 different signal transduction and Protein-Protein interaction database for that purpose (See Table S1 of Text S1), but unfortunately due to proper maintenance and lack of database update procedure from time to time, there was no database that could give us the most up to date and comprehensive pathway map. As mentioned earlier, the main problem was collation of data from different database which shows heterogeneity of information. The number of proteins and their interactions presented in these databases was not equal and due to poor annotation method used by different database caused major obstacle to make a comprehensive pathway map of Hedgehog. Our comparative analysis of the Hedgehog pathway data provided in few important existing databases proves this fact (See Table S2 of Text S1). though the basic structure of the pathway map were obtained from the data collated in different database, but in order to make a most up to date and reliable pathway map with more number of proteins and interactions, published scientific literatures and experimental studies were consulted. We mainly used “Google Scholar” and “Pubmed” to search these literatures. The abbreviation and documentation of the proteins of our newly reconstructed Hedgehog pathway are provided in Table of Text S1with proper literature and database references. More than 50 published papers and 21 databases were studied to collate the relevant information for Hedgehog pathway.

This reconstructed pathway map (Figure 1) was a master model that accounts for all the possible proteins and their interactions reported in different cell types across different experimental conditions. The maps provided in Figure 1 and Figure 2 are only based on human cell line specific data, but do not indicate any particular cell type or disease specific scenario. The reason for the construction of such global maps was to consolidate up to date information about the pathway for further analysis and also the formation of a platform that could be used for further exploration of this pathway. This map included all the probable proteins and interactions that govern the flow of the signal, from input to intermediate to output layer. The map was created using CellDesigner Ver. 4.2. [29], a software package that allows users to depict molecular interactions using standard systems biology notations. Using the GUI based drawing tool of this software, we attempted to capture or draw most of the significant interactions of this pathway along with their cross talks. We included only those proteins and interactions in our reconstructed map that have at least one human cell specific experimental evidence in any published research article. On the basis of this criterion we were able to include more number of proteins and interactions to the newly reconstructed map (Figure 1). This extensive data mining from literature and database sources provided us the sufficient data to reconstruct a new and more informative hedgehog signaling map of human.

### Graph and Parameters

In order to convert the whole hedgehog signaling pathway into a graph or network model [92], at first we constructed an adjacency symmetric matrix ‘*A*’, where rows and columns were the proteins of the pathway. In this graph the proteins were the ‘nodes’ and the direct connection (flow of signal or interaction) between two proteins were considered as an ‘edge’. The graph was a ‘*directed graph*’ or ‘*digraph*’. That means a connection between two nodes had a specific direction. The following condition was used to construct the adjacency matrix ‘*A*’. Let, *A_ij_* be the element of *i* ^th^ row and *j* ^th^ column of the adjacency matrix ‘*A*’, then *A*_ij_ **= **{**1**: If species *i* is interacting or transmitting the signal to *j*; **0**: If *i* and *j* have no connection; **−1**: If species *j* is interacting or transmitting the signal to *i*}.

The parameters “In-Degree”, “Out-Degree”, “Total Degree”, “Betweenness Centrality”, “Closeness Centrality”, and “Eigenvector Centrality” were calculated on the basis of the adjacency matrix A. Then we made a network file in ‘.net’ format (default input format of the network manipulation and visualization software ‘Pajek’ [93], See the directory “GRAPH_PROJECT” of File S1) for a directed graph from this matrix and used it in Gephi 0.8.1 [94] to calculate all types of Degree and Eigenvector centrality. The network figure (Figure 2) was also drawn in Gephi. We also used the.net file in ‘igraph’ [95] (a software package of ‘R' [96]) to calculate “All pairs shortest paths” and in Pajek to calculate the Betweenness and Closeness centrality. In order to visualize all pairs shortest paths, (Figure S2) we used Matlab® (R2012b, The MathWorks) sparse matrix function ‘Spy’. We also used ‘Microsoft Excel (2007)’ for the general plotting purpose.

A brief description of the parameters used in our study is given below:

#### In-Degree (*K_in_*)

It refers the total number of nodes (activations or inhibitions) that are directly acting on a particular node in the network [92].

#### Out-Degree (*K_out_*)

The total number of interactions (activations or inhibitions) that are acting by a particular node on the other nodes in the network [92].

#### Degree (*K_i_*)

It refers the total number of in-degree and out-degree of a particular node [81]. Therefore, total degree (***K_i_***) of a node ‘***i*** *’* is calculated as.
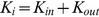
(1)


#### Eigenvector centrality

It refers that a node in a network will be more central if it is connected to many central nodes in the network [46], [47]. According to Newman [97], the centrality ***x_i_*** of a node ***i*** is directly proportional to the cumulative sum of the centralities of its neighbors’ ***x_j_***.
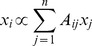
(2)

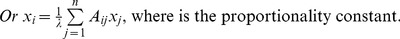
(3)


Now, if we consider the above equation (3) as a vector equation, then we can write that
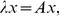
(4)where, *x = (x_1_, x_2_, x_3_……x_n_)* is the Eigenvector of the adjacency matrix *A* having highest positive Eigenvalue *λ.*

#### Betweeness centrality

It is the ratio of the number of shortest paths that pass through the node to the total number of shortest paths of all the nodes to all the other nodes. It signifies that how a node is important in the shortest paths of all the other nodes of the network [47].

#### Closeness centrality

The Closeness centrality of a node is defined as the inverse of sum of the total length of the distances or shortest paths of that node to the other nodes [47]. Therefore higher closeness centrality of a node implies the lower length of shortest paths to the all other nodes in the network and signifies how close a node is situated from the other nodes in the network [47].

#### Shortest path (*L_ij_*)

It refers the minimum number of intermediate links or connections that have to traverse from one node ‘*i’* to the another node ‘*j’* [48].

### Logical Modeling

The entire Hedgehog signaling network was organized into a three layered system of input, intermediate and output, with input signals orchestrating cellular responses to output via intermediate molecules. To visualize and analyze the Hedgehog signal transduction network, we constructed the Logical or Boolean Interaction Hyper-graph with large number of nodes and interactions or hyper-arc [1]. In our Boolean network each node represented a protein (Ligands, Receptors, kinase or Transcription factor) or cellular response (Cell Proliferation, Cell cycle progression, Wnt Pathway etc.) whose state can be either be 0 (OFF) or 1 (ON). Depending on the cellular function and/or location, the proteins may be active (ON) or inactive (OFF). The entire simulation of Boolean modeling was performed in CellNetAnalyzer [49]-[51] and the following steps were followed during the logical simulation.

#### i) Selection of input and output proteins

In order to construct a logical model for hedgehog signaling network, at first we considered the input and output nodes. Mainly the proteins, which did not have upstream connection in the reconstructed pathway map (Figures 1 and 2), were considered as input proteins. Similarly, the proteins, which were the downstream effectors of input proteins, were considered as output proteins in our model. Though there were few exceptions in our model while considering the input proteins for three cancer scenarios (Glioma, Colon and Pancreatic) with respect to the normal pathway scenario. In that case, we considered the three ligands SHH, IHH and DHH as inputs, though they had the upstream connections. In order to simulate their over expression effects in the cancer scenarios, we kept their constant active state as 1 or ON throughout the simulations. But in normal scenario, we considered their expression or logical state according to the state of their upstream activators (e.g. BMP_RUNX3, CDO, BOC etc.). The names of the input and output proteins was provided in Table S3 of Text S1 with proper documentation. The logical states of the input proteins were considered from various literature sources (See Table S4 of Text S1), EBI-ArrayExpress Atlas [98] and also from various signaling and cancer databases (See Table S1 of Text S1).

#### ii) Construction of Boolean or logical equations

We know that the molecular species of a biological signaling network is highly interconnected and interdependent with each other. Using this phenomena one can easily construct Boolean or Logical equations that could show their inter relationship with each other within the network. We manually formed the Boolean equations among all the nodes of the hedgehog signaling network using our biological understanding of the interactions from various literature sources. The entire list of the Boolean equations is available in Table S5 of Text S1. In this table we also provided the procedures that we followed to construct a Boolean equation with few examples. There were total 96 Boolean equations and 63 nodes including cellular responses presented in our model that was used to simulate the hedgehog pathway in CellNetAnalyzer [49]–[51]. This entire set of Boolean equations was our “Master Boolean Model” (See the directory “CNA_PROJECT” of File S1), which we used to simulate different scenarios by varying the logical states of the input proteins.

#### iii) Simulation

Using the master model of Boolean equations, we performed our simulation analysis in CellNetAnalyzer [49]–[51]. The entire simulation was done using a logical framework described extensively in previous studies [49]–[51]. In order to create different scenarios, we altered the logical states (“0” as “OFF” or “1” as “ON”) of the input proteins. We considered both the ‘gain-of-function’ (“ON” or “1”) states of the oncogenic proteins like RAS, ERK12, TWIST etc., and the “loss of functions” (“OFF” or “0”) of few tumor suppressor proteins like GAS1, SUFU, NUMB, SNO while creating different cancer scenarios in the logical simulation (See Table S4 of Text S1). As it is known that not only the over expression of some proteins can cause cancer but several tumor suppressor proteins in the network are also lost during cancer progression, hence this perturbation helps to test the model from both perspectives. Also, in order to simulate the scenarios in temporal space, we accounted the time scales into the Boolean equations of our master model. As we know that in a signaling network few reactions happen later than some other reactions, therefore there was a need to imply “Time Scale” in this Boolean analysis. In our model there were three reactions (production of GLI1, PTCH1 and HHIP) that actually occur as feedback loops in the network. In order to discriminate these reactions, we kept them in “time scale 2” irrespective of the rest of the reactions in “time scale 1”. Also, the productions of output proteins from nuclear region were considered in time scale 2. The proteins which presented in different locations (e.g. GLI1) in the pathway map (Figure 2) were named according to their cellular location (e.g. NUC_GLI1 where NUC stands for Nuclear) and considered as two different nodes in the model.

After performing the “time scale 2” simulation for each scenario, the entire simulation results provided in “Results” section and the logical steady states of the output proteins in Table S4 of Text S1 were considered. At the time of calculation, the upstream and downstream effectors from the “Dependency matrix” of the nodes for each scenario, we excluded the reactions with given zero values in CellNetAnalyzer [49]–[51]. These Dependency matrices for each scenario were then used to calculate the total number of “Upstream” and “Downstream” effectors of each node in the network.

#### iv) Identification and perturbation of important proteins

In order to identify the important proteins which were responsible for a particular cancer, we compared the total number of upstream effectors and downstream effected proteins of each protein of that cancer scenario with each protein of normal scenario. From these comparisons we identified the proteins that were showing significant variation in cancer scenarios with respect to the normal scenario. We then extracted those identified proteins from each cancer scenarios and this helped us to identify the sole or combination of target proteins for the perturbation analysis. Among these identified proteins we selected few minimal combinations of target proteins in perturbations analysis, based on the results from Graph theoretical analysis and also biological feasibility of targeting these proteins in cancer cell. Finally we choose these minimal combinations of such proteins for each cancer scenarios that could be perturbed in the *in-silico* analysis, where the logical states of these selected target proteins were altered and the same simulation analysis as discussed in *step iii* were performed.

A brief description of a few logical formalisms used in our study is given below:

#### i) Hyper-arc & hyper-graph

An interaction hyper-arc is a set of the arcs which connects more than one node to a particular single node. Therefore, a hyper-arc may contain more than one node at its source end (input) but one node at its sink end (output). A hyper-graph (*H*) consists of the set of hyper-arcs (*A*) and the set of nodes (*N*) present in the network or graph [49]. In our case the hyper-graph is directed hyper-graph *H = (N, A)* as the edges or arcs of the proteins of hedgehog signaling network have unique direction.

#### ii) Feedback loop and time scale

In several network it has been found that the product of a reaction is actually producing the reactant or interacting back with one of the reactants of the reaction. This type of connections or arcs or edge is basically form a “loop” in the network and as the output comes back again to its input or source nodes, hence this type of connection is called feedback loop. Feedback loop may be positive or negative depending on the nature of its connection. In our hedgehog signaling model, we have found that GLI1 helps to activate GLI3_A and as a result GLI3_A produces GLI1. This is an example of positive feedback loop whereas production of PTCH1 or HHIP from GLI1 or NUC_GLI1 is an example of Negative feedback loop. It is also worth mentioning that to perform these kinds feedback analysis one should assign time scale to the reactions where feedback loop reactions would be in higher time scale compare to the reactions executed by the inputs.

#### iii) Logical steady states, synchronous and asynchronous update

When the logical states of the nodes reach to a certain constant or steady state value with the state of the associated logical functions or equations, such that the switching of logical values of the nodes stop during infinite time scale, then such state is called logical steady state.

The updates of the logical states of the nodes in a Boolean network can be updated synchronously or asynchronously. In the Synchronous model, the logical state *S_i_ (t)* of a node *‘I’* at time scale *‘t’* is updated to the next time scale *‘t+1’* by a Boolean function ‘*B*’, in such a way that *S_i_ (t+1) = B {S_i_ (t)}*. On the other hand, in Asynchronous model the logical state of a node *‘I’* is kept fixed, so that its current logical state is unequal to its associated Boolean function, *i.e. S_i_ (t+1) ≠ B {S_i_ (t)}* [49].

We simulated our model using both the synchronous and asynchronous updating approaches in Odefy (software package used in CellNetAnalyzer [49]–[51]), but after attaining at the logical steady state we found that both the simulation results were showing same dynamics (results not shown), since no further changes in the states of the nodes can be observed once it reaches the logical steady state [49]. In our logical model, to create the cancer scenarios and to observe the effect of over expression of the ligands in cancer development, we used asynchronous update of SHH, IHH and DHH by considering their logical input state as “1” or “ON” throughout whole simulation time points (*i.e.*Time point 1 and 2).

#### iv) Dependency matrix

It is a matrix (*D*) whose rows (*i*) and columns (*j*) are the nodes and each cell (*D_ij_*) defines the logical relationship between a pair of node. This dependency matrix is helpful to find the activator or inhibitor molecules of a node or the molecules which are activated or inhibited by a particular node in the network.

#### Model validation

The simulation results shown for all three types of cancer was done through in-silico logical analysis, but in order to validate our model with cancer cell line specific experimental data, we considered the microarray expression data (UP or DOWN regulated) of the proteins for Glioma Grade IV (accession numbers GSE4290) and Pancreatic cancer cell line (accession numbers GSE16515) from EBI ArrayExpress Atlas [98]–[100]. Unfortunately, we could not found any suitable expression data of colon cancer cell line as the expression of most of the proteins were showing not significant or high *P*-value. In this case, we considered the expression level of five hedgehog proteins: SHH, PTCH1, HHIP, GLI3 or GLI3_Active and PDGFRA, which were detected by using RT-PCR, in-situ hybridization and immuno-histochemistry experiments by Yue-Hong Bian et al. [101].

The expression levels of few proteins from both the microarray data were not found significant as their corresponding *P*-value was greater than 0.005. We kept the expression of these proteins as undetermined. For better biological interpretations of the model predictions, the model was then simulated in two ways to validate with these experimental observations for three types of cancers. In Simulation 1, we considered the logical states mentioned earlier in “Simulation” section and in Table S4 of Text S1. In Simulation 2, we considered the expression of input proteins observed from the experimental data for each of these cancer types. Since it is known that the activity of CKI_A to down regulate GLI proteins in cytoplasm requires BTRCP, PKA_ALPHA, and GSK3 [35], [36], hence at the time of Simulation 2, we altered the expression level of CKI_A and ITCH from UP (1) to Down (0) and obtained the expression values. This consideration was not available in the particular experimental data considered to validate our model. Otherwise we were able to match the down regulation of GLI proteins as well as some output proteins like BMI, BCL2, SNAI1 etc., which showed up regulation in experimental data. The expression values of the input proteins used in simulation 1 were taken from Table S4 of Text S1. We also performed another simulation (i.e. Simulation 2) by considering the expressions of the proteins determined in microarray expression data. While doing this simulation, we altered the expressions of CKI_A in cytoplasm region and NUMB, ITCH in nuclear region. In microarray expression data, we found their up regulation but as we had taken these proteins as repressors of GLI transcription factors in our model (Table S5 of Text S1), therefore we considered their logical expressions as ‘0’, so that we can examine their effect of “loss of function” in pancreatic cancer cell line. By considering the Up regulation or logical state ‘1’ of these proteins in our model, we could not see the up regulation of GLI and other output onco-proteins of our pancreatic cancer model. A comparative statistics of the percentage of accuracy between experimental and simulation results of each cancer scenario was presented in Table S6 of Text S1.

## Supporting Information

Figure S1 **Venn diagram representing comparison of number of proteins between other database and our model.** This Venn diagram represents a comparative view of number of proteins in our model with existing major databases, KEGG, BIOCARTA, GENE GO, NETPATH and PATHWAY CENTRAL, considered to reconstruct the Hedgehog pathway diagram (Figure 1 and Figure 2). The overlapping regions between two circles (i.e. two databases or anyone of the database and our model) are representing the same proteins which have mentioned in both the databases. The large non-overlapping area shown by OUR MODEL signifies the information of the large number of proteins which were not found in anyone of the above mentioned databases and are taken from other literature sources.
(TIF)

Figure S2 **All pairs shortest paths of the proteins of Hedgehog signaling network.** The values of shortest path(s) between two proteins in the Hedgehog signaling network (shown in Figure 2) is presented with the name of the proteins arranged in both row and column wise. Different colors are used to distinguish the different values of shortest path. White cells represent zero value or no shortest path. The lower part (i.e. from Cyclin_B to Cyclin_D2) corresponds to the Output proteins of Hedgehog pathway and hence there are no connections of these proteins with the remaining proteins in network (Figure 2).
(TIF)

Figure S3 **Probability distributions of the Shortest paths of Hedgehog signaling network.** The X-axis represents the value of the shortest paths from 1 to 8 and Y-axis represents the probability of getting a particular shortest path in the network. The shortest path ‘3’ has highest probability in the distribution and the average shortest path is calculated as 3.581.
(TIF)

Figure S4 **Dependency matrix of SMO inhibition scenario in Glioma model.** The X and Y axes represent the name of the proteins of our Hedgehog signaling model. This figure shows the interdependency between a pair of proteins (Activators in green and Inhibitors in red) in Glioma model after SMO inhibition (marked by black arrow). Most of the upstream activators of GLI1, GLI2, GLI3_A such as HFU, ULK3, RAS, TWIST, ERK12 and other hedgehog responsive oncoproteins are still present in the simulation results.
(TIF)

Figure S5 **Dependency matrix of SMO inhibition scenario in Colon cancer model.** The X and Y axes represent the name of the proteins of our Hedgehog signaling model. This figure shows the interdependency between a pair of proteins (Activators in green and Inhibitors in red) in Colon cancer model after SMO inhibition (marked as black arrow). Most of the upstream activators of GLI1, GLI2, GLI3_A such as HFU, ULK3, RAS and other hedgehog responsive oncoproteins are still present in the simulation results.
(TIF)

Figure S6 **Dependency matrix of SMO inhibition scenario in Pancreatic cancer model.** The X and Y axes represent the name of the proteins of our Hedgehog signaling model. This figure shows the interdependency between a pair of proteins (Activators in green and Inhibitors in red) in Pancreatic cancer model after SMO inhibition (marked as black arrow). Most of the upstream activators of GLI1, GLI2, GLI3_A such as HFU, ULK3, RAS, and ERK12 and also the other hedgehog responsive oncoproteins are still present in the simulation results.
(TIF)

Text S1 **Supplementary text.** This supplementary text contains five supplementary tables (Table S1–S6) referred in the main article and formulation of the Logical equations.
(DOCX)

File S1 **Model files used to simulate the structural and logical analysis of the normal scenario.** This zip file contains the files which were used to simulate the structural and logical analysis of Hedgehog signaling pathway in normal scenario. There are two directories within this zip file: “CNA_PROJECT” and “GRAPH_PROJECT”. The directory “CNA_PROJECT” contains the Cell Net Analyzer project files to simulate the Normal hedgehog pathway scenario using initial logical states provided in “normal.val” file. One can also create the other scenarios by varying the logical states of the proteins. On the other hand the directory “GRAPH_PROJECT” contains a.net file which can be used to calculate the graph theoretic parameter values using Gephi or Pajek software.
(RAR)
