# Supplementary figures and images for: Structural and Logical Analysis of a Comprehensive Hedgehog Signaling Pathway to Identify Alternative Drug Targets for Glioma, Colon and Pancreatic Cancer
Source: PLoS One. 2013 Jul 23;8(7):e69132. doi: 10.1371/journal.pone.0069132 (PMC3720582; doi:10.1371/journal.pone.0069132)

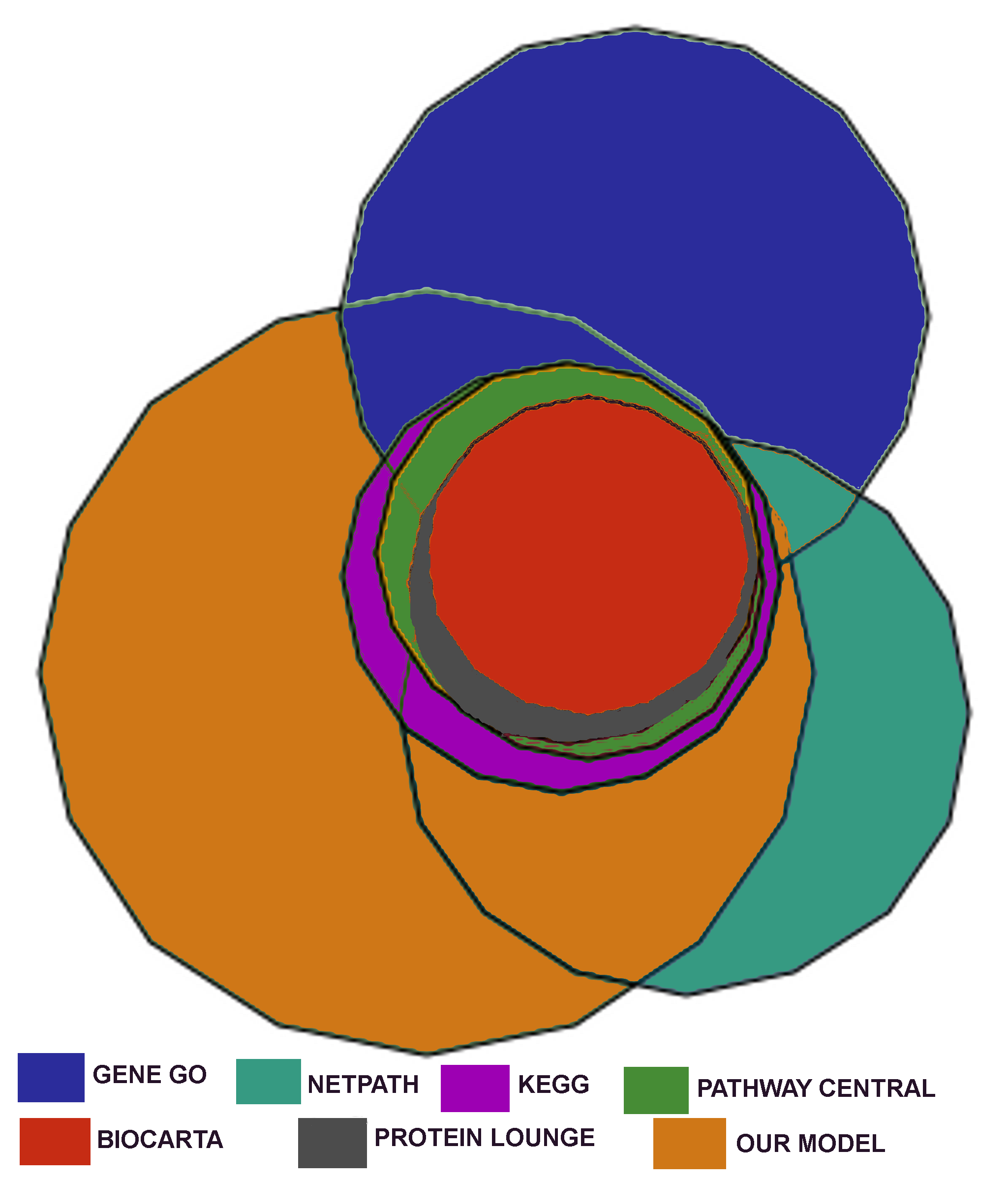

Supplement: Figure S1 — Venn diagram representing comparison of number of proteins between other database and our model. This Venn diagram represents a comparative view of number of proteins in our model with existing major databases, KEGG, BIOCARTA, GENE GO, NETPATH and PATHWAY CENTRAL, considered to reconstruct the Hedgehog pathway diagram (Figure 1 and Figure 2). The overlapping regions between two circles (i.e. two databases or anyone of the database and our model) are representing the same proteins which have mentioned in both the databases. The large non-overlapping area shown by OUR MODEL signifies the information of the large number of proteins which were not found in anyone of the above mentioned databases and are taken from other literature sources. (TIF) [file pone.0069132.s001.tif]

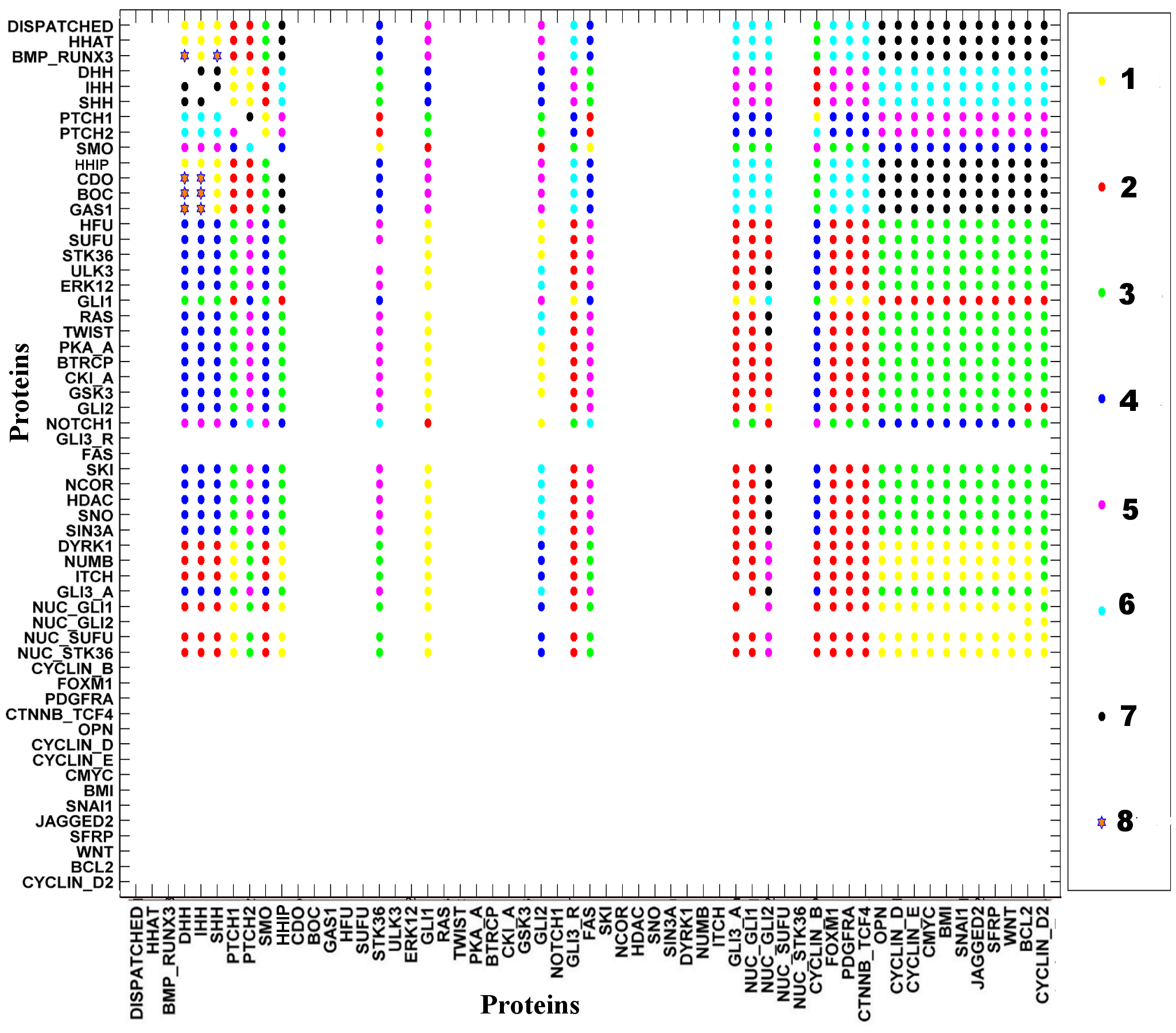

Supplement: Figure S2 — All pairs shortest paths of the proteins of Hedgehog signaling network. The values of shortest path(s) between two proteins in the Hedgehog signaling network (shown in Figure 2) is presented with the name of the proteins arranged in both row and column wise. Different colors are used to distinguish the different values of shortest path. White cells represent zero value or no shortest path. The lower part (i.e. from Cyclin_B to Cyclin_D2) corresponds to the Output proteins of Hedgehog pathway and hence there are no connections of these proteins with the remaining proteins in network (Figure 2). (TIF) [file pone.0069132.s002.tif]

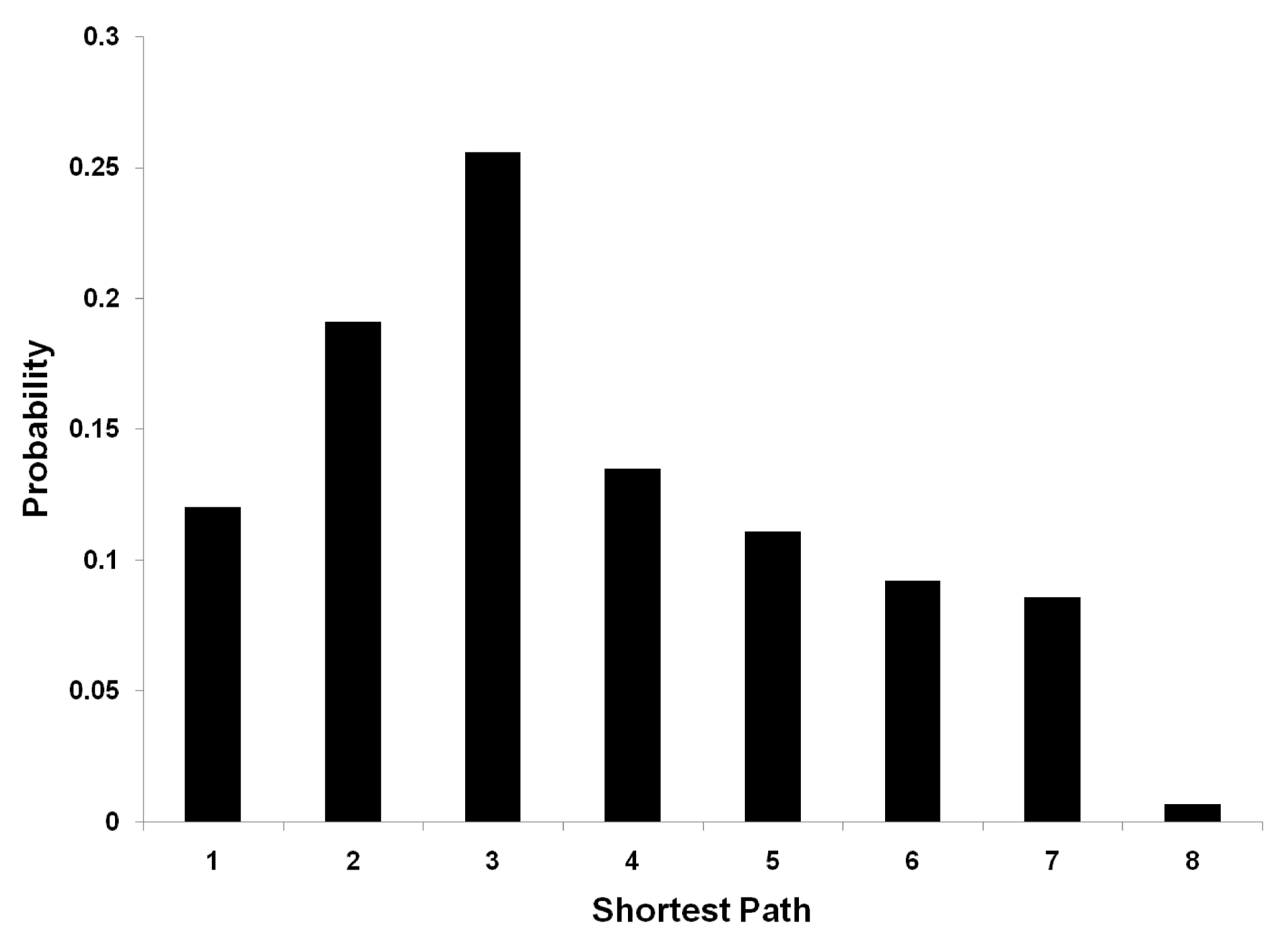

Supplement: Figure S3 — Probability distributions of the Shortest paths of Hedgehog signaling network. The X-axis represents the value of the shortest paths from 1 to 8 and Y-axis represents the probability of getting a particular shortest path in the network. The shortest path ‘3’ has highest probability in the distribution and the average shortest path is calculated as 3.581. (TIF) [file pone.0069132.s003.tif]

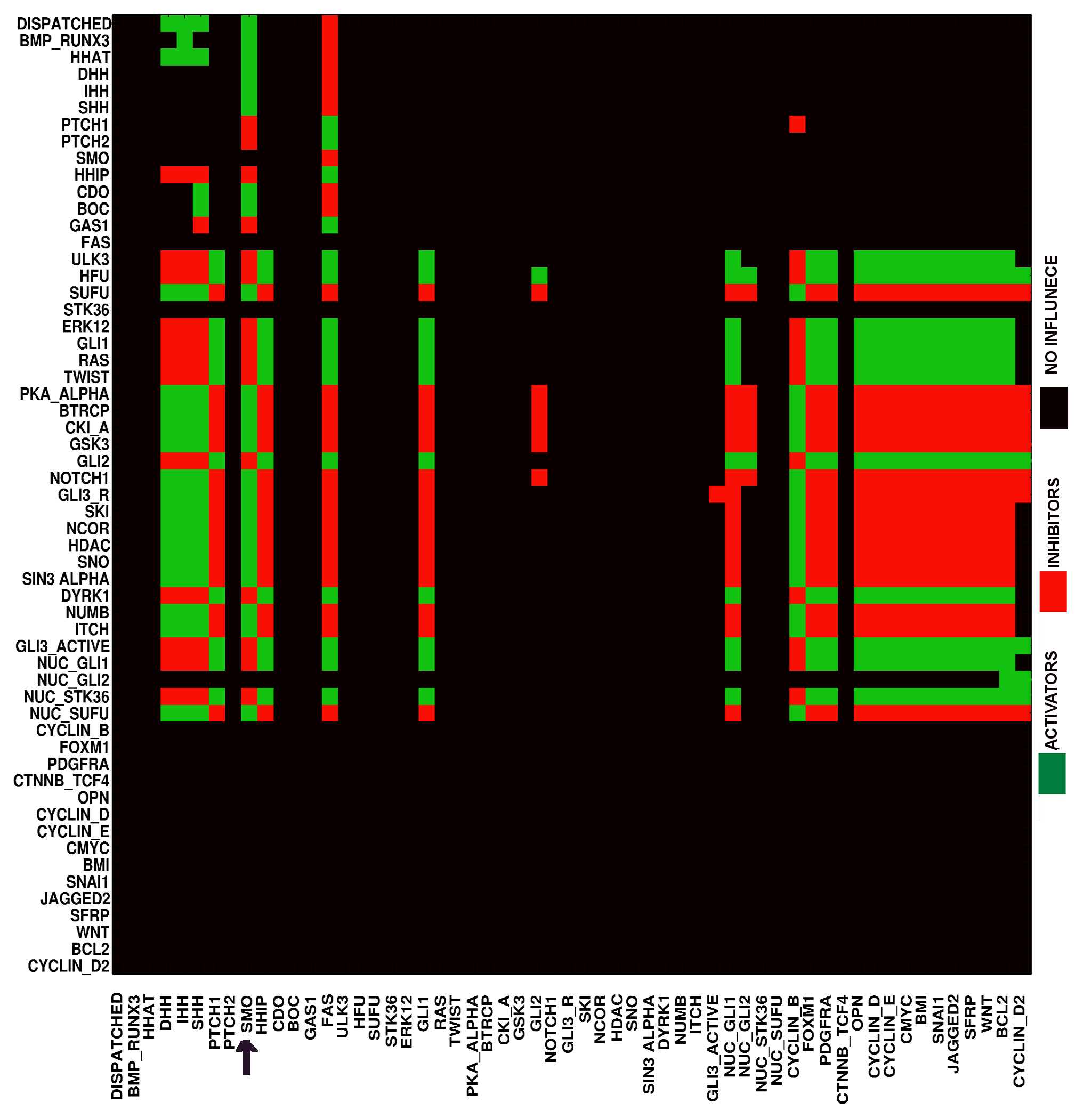

Supplement: Figure S4 — Dependency matrix of SMO inhibition scenario in Glioma model. The X and Y axes represent the name of the proteins of our Hedgehog signaling model. This figure shows the interdependency between a pair of proteins (Activators in green and Inhibitors in red) in Glioma model after SMO inhibition (marked by black arrow). Most of the upstream activators of GLI1, GLI2, GLI3_A such as HFU, ULK3, RAS, TWIST, ERK12 and other hedgehog responsive oncoproteins are still present in the simulation results. (TIF) [file pone.0069132.s004.tif]

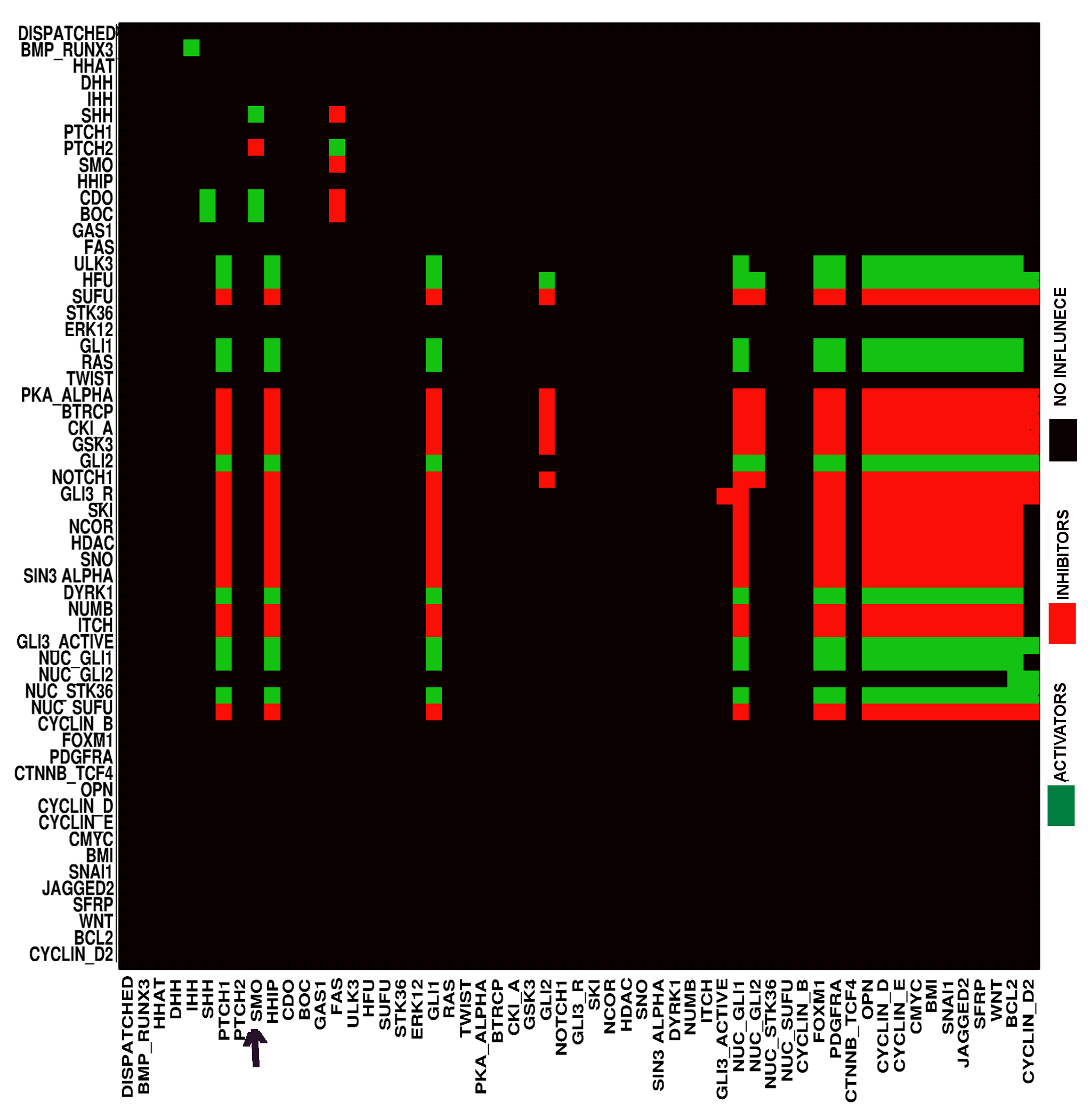

Supplement: Figure S5 — Dependency matrix of SMO inhibition scenario in Colon cancer model. The X and Y axes represent the name of the proteins of our Hedgehog signaling model. This figure shows the interdependency between a pair of proteins (Activators in green and Inhibitors in red) in Colon cancer model after SMO inhibition (marked as black arrow). Most of the upstream activators of GLI1, GLI2, GLI3_A such as HFU, ULK3, RAS and other hedgehog responsive oncoproteins are still present in the simulation results. (TIF) [file pone.0069132.s005.tif]

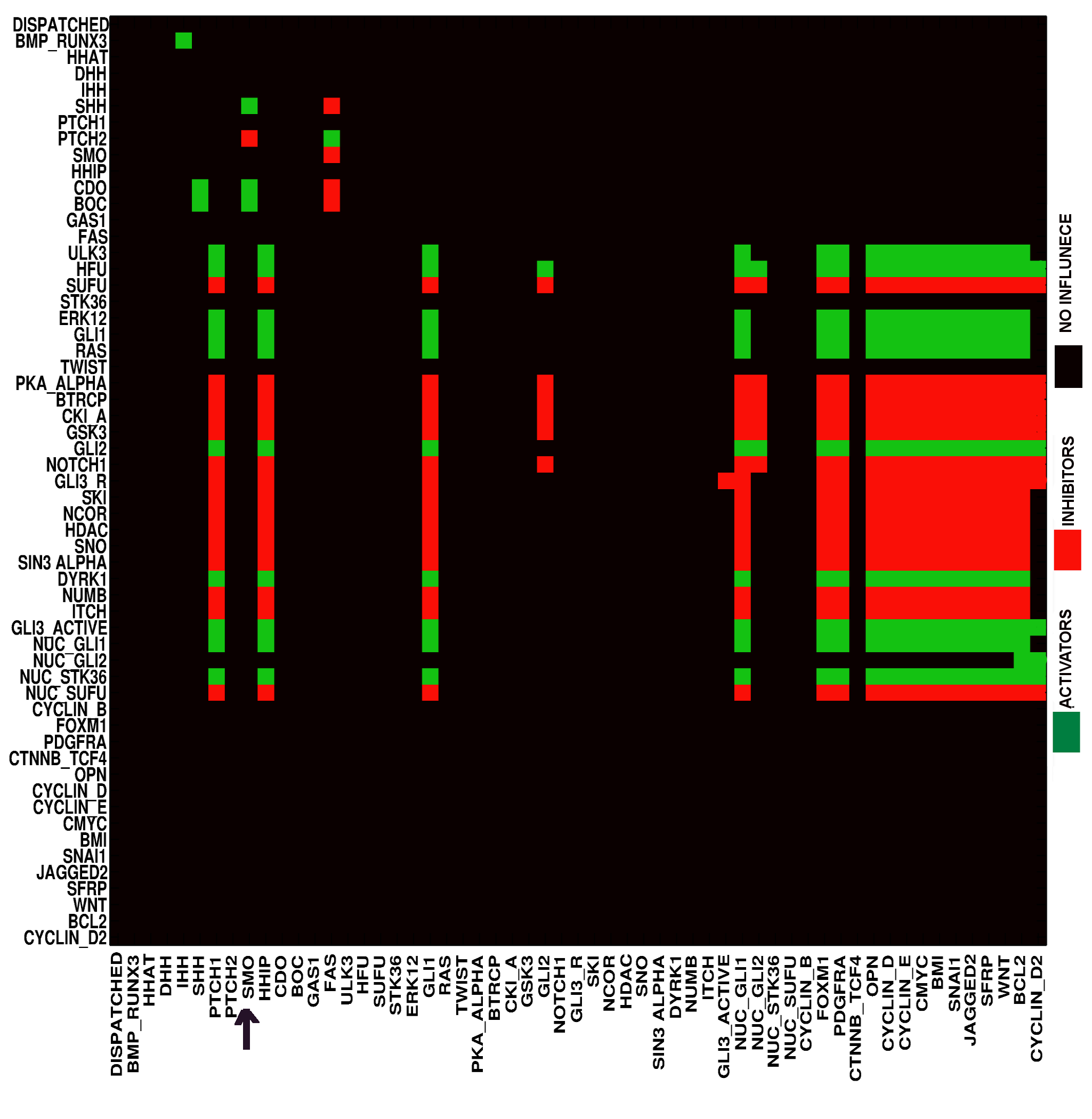

Supplement: Figure S6 — Dependency matrix of SMO inhibition scenario in Pancreatic cancer model. The X and Y axes represent the name of the proteins of our Hedgehog signaling model. This figure shows the interdependency between a pair of proteins (Activators in green and Inhibitors in red) in Pancreatic cancer model after SMO inhibition (marked as black arrow). Most of the upstream activators of GLI1, GLI2, GLI3_A such as HFU, ULK3, RAS, and ERK12 and also the other hedgehog responsive oncoproteins are still present in the simulation results. (TIF) [file pone.0069132.s006.tif]
