# Supplementary material for: Structural and Logical Analysis of a Comprehensive Hedgehog Signaling Pathway to Identify Alternative Drug Targets for Glioma, Colon and Pancreatic Cancer
Source: PLoS One. 2013 Jul 23;8(7):e69132. doi: 10.1371/journal.pone.0069132 (PMC3720582; doi:10.1371/journal.pone.0069132)
Supplement: Text S1 — Supplementary text. This supplementary text contains five supplementary tables (Table S1–S6) referred in the main article and formulation of the Logical equations. (DOCX) [file pone.0069132.s007.docx]

**Text S1: Supplementary Text**

**Table S1. Name of the Database used to collate information for reconstructing the Hedgehog Pathway**

| **Sr. No.** | **Name of the Database** | **Available at** |
| --- | --- | --- |
| 1. | KEGG | http://www.genome.jp/kegg/pathway.html |
| 2. | WikiPathways | http://www.wikipathways.org/index.php/WikiPathways |
| 3. | Millipore | http://www.millipore.com/pathways/pathviewer.do?pathwayId=163 |
| 4. | Invitrogen | http://products.invitrogen.com |
| 5. | Reactome | http://www.reactome.org/ReactomeGWT/entrypoint.html |
| 6. | HPRD | http://www.hprd.org |
| 7. | Netpath | http://www.netpath.org/ |
| 8. | APID | http://bioinfow.dep.usal.es/apid/index.htm |
| 9. | Biocompare | http://www.biocompare.com |
| 10. | GeneGo | http://www.genego.com |
| 11. | Applied Biosystem | http://www5.appliedbiosystems.com/tools/pathway |
| 12. | Pathway Central | http://www.sabiosciences.com/pathway.php?sn=Hedgehog |
| 13. | CNPD | http://cpdb.molgen.mpg.de/ |
| 14. | Cell Signaling Technology | http://www.cellsignal.com/ |
| 15. | Pathway Studio | http://www.ariadnegenomics.com/products/pathway-studio |
| 16. | Biocarta | http://www.biocarta.com/genes/index.asp |
| 17. | Biomodels | http://www.ebi.ac.uk/biomodels-main |
| 18. | PID-NCI | http://pid.nci.nih.gov |
| 19. | CancerCellMap | http://cancer.cellmap.org/cellmap/ |
| 20. | EBI-ARRAYEXPRESS | http://www.ebi.ac.uk/arrayexpress/ |
| 21. | PathwayCommons | http://www.pathwaycommons.org |

**Table S2. Comparative statistics of number of species and interactions for Hedgehog pathway in different database**

| **Database** | **Number of Species present** | **Number of Species taken in our model** | **Number of Interactions** |
| --- | --- | --- | --- |
| KEGG | 18 | 18 | 13 |
| Protein Lounge | 12 | 12 | 14 |
| Biocarta | 10 | 10 | 15 |
| Netpath | 31 | 14 | 57 |
| GenGo | 33 | 8 | 43 |
| Pathway Central | 14 | 14 | 14 |
|  |  |  |  |
| **Reconstructed Hedgehog Pathway in this study** | **57** | | **96** |

**Table S3. Abbreviations and Detail information about the Proteins and Cellular Responses involved in the newly reconstructed Hedgehog pathway**

| **SHORT NAME USED IN MODEL** | | **FULL NAME** | **DOCUMENTATION** |
| --- | --- | --- | --- |
| **I. Extracellular and Membrane Proteins** | | | |
| BMP_RUNX3 | Bone Morphogenetic Protein and Runt related transcription Factor 3 | | BMP-RUNX3 signaling induces expression of IHH in surface differentiated epithelial cells of stomach and intestine. |
| DISPATCHED | Dispatched | | Dispatched regulates the release and extracellular accumulation of cholesterol-modified hedgehog proteins and is hence required for effective production of the Hedgehog signal. |
| HHAT | Hedgehog Acyltransferase | | HHAT is a hedgehog modifier which induces lipid modification to generate mature peptides. Hedgehog proteins with lipid modification are then released from producing cells by Dispatched homologues. |
| DHH | Desert Hedgehog | | Three Hedgehog ligands (homologues proteins) of Hedgehog pathway considered as Input Proteins in this model. |
| IHH | Indian Hedgehog | |  |
| SHH | Sonic Hedgehog | |  |
| PTCH1 | Patched1 | | Two homologue of receptor protein Patched. In the absence of hedgehog ligands these proteins inhibit another trans membrane protein Smoothened (SMO). |
| PTCH2 | Patched2 | |  |
| SMO | Smoothened | | G-protein coupled receptor that is normally suppressed by Patched receptors but is activated in the presence of Hedgehog ligands (SHH, DHH, IHH). |
| HHIP | Hedgehog Interacting Protein1 | | Regulates the amount of Hedgehog ligand that can bind to Patched receptors |
| CDO | Belong to Immunoglobin super family. | | CDO and BOC represent a subfamily within the Ig super-family, consisting of an ectodomain comprised of four (BOC) or ﬁve (CDO) Ig repeats, followed by three ﬁbronectin type III (FNIII) repeats and a long, divergent intracellular domain. |
| BOC | Brother of CDO. | |  |
| GAS1 | Growth arrest specific gene | | Regulates the amount of Hedgehog ligand that can bind to Patched receptors along with HHIP. |
| **II. Cytoplasmic Proteins** | | | |
| HFU | | Human Fused | Stimulates GLI1 and GLI2 transcription factors. |
| SUFU | | Suppressor of fused homolog | Sequesters GLI proteins in the cytoplasm and prevents tarnscription of target genes. |
| STK36 | | Serine/threonine-protein kinase 36 | Up-regulation of GLI transcription activity. |
| ERK12* | | Extracellular signal-regulated kinase | Up-regulation of GLI transcription activity. |
| GLI1 | | Transcriptional activator Gli1 | Mediates target gene expression. |
| GLI2 | | Transcriptional activator Gli2 | Mediates target gene expression. |
| GLI3_R | | Transcriptional repressor Gli3 | Antagonises target gene expression byother Gli factors. |
| RAS* | | Ras protein (GTPase activity) | RAS and TWIST activate GLI1 regulatory sequences. |
| TWIST* | | Twist-related protein | Is known to activate GLI1. |
| PKA_A | | Protein Kinase alpha | Phosphorylates and activates SMO. |
| BTRCP | | Beta-transducin repeat-containing protein | Involoved in ubiquitination of Gli1 resulting in the formation of a transcriptional repressor. |
| CKI_A | | Casein Kinase I isoform alpha | Known to elicit negative effects on GLI |
| GSK3 | | Glycogen synthase Kinase 3 | Known to elicit negative effects on GLI |
| NOTCH1* | | Notch1 protein | Known to elicit negative effects on GLI |
| FAS* | | Apoptosis-mediating surface antigen FAS | Mediates apoptosis |
| ULK3 | | Unc-51-like kinase 3 | Serine Threonine kinase present in addition to STK36 that functions in up-regulation of GLI transcriptional activity |
| **III. Nuclear Proteins** | | | |
| NUC_GLI1 | | Nuclear GLI1 | Represents the nuclear form of GLI1 protein. |
| NUC_GLI2 | | Nuclear GLI2 | Represents the nuclear form of GLI2 protein. |
| NUC_SUFU | | Nuclear SUFU | Represents the nuclear form of SUFU. |
| NUC_STK36 | | Nuclear STK36 | Represents the nuclear form of STK36 |
| GLI3_A | | Activated GLI3 for Transcription | Mediates target gene expression |
| SKI | | Proto-oncogene C-Ski | Functions as a transcriptional co-repressor |
| NCOR | | Nuclear receptor corepressor | Functions as a transcriptional co-repressor |
| HDAC | | Histone deacetylase | Functions as a transcriptional co-repressor |
| SNO | | Ski-like protein or Ski-related oncogene | Functions as a transcriptional co-repressor |
| SIN3A | | Paired amphipathic helix protein Sin3 alpha | Functions as a transcriptional co-repressor |
| DYRK1 | | Dual Specificity Tyrosine phosphorylation Regulated Kinase 1A / Dual Specificity Yak1 related Kinase | Known to substantially increase GLI mediated transcription |
| NUMB | | Protein numb homolog | Numb along with ubiquitin ligase such as Itch is able to polyubiquitinate GLI1 and target it for degradation and thus control HH signaling. |
| ITCH | | E3 ubiquitin-protein ligase Itchy homolog |  |
| **IV. Output Proteins** | | | |
| CTNNB_TCF4 | | Nuclear form of TCF4 | Represents the nuclear form of TCF4 |
| CYCLIN_B | | G2/mitotic-specific cyclin-B1 | Mediates cell cycle regulation |
| CYCLIN_D | | G1/S-specific cyclin-D | Mediates cell cycle regulation |
| CYCLIN_D2 | | G1/S-specific cyclin-D2 | Mediates cell cycle regulation |
| CYCLIN_E | | G1/S-specific cyclin-E | Mediates cell cycle regulation |
| FOXM1 | | Forkhead box protein M1 | Implicated in cellular proliferation |
| PDGFRA | | Platelet Derived Growth Factor receptorisoform alpha | Transmembrane receptor |
| OPN | | Osteopontin | Osteopontin is a secreted protein that influences multiple downstream signaling events that allow cancer cells to resist apoptosis, evade host immunity and influence growth of indolent tumors. |
| CMYC | | Myc proto-oncogene protein | Mediates cellular proliferation. |
| BMI | | Polycomb complex protein BMI-1 | BMI-1 is a transcriptional repressor belonging to the polycomb gene family and its suppressor functions are involved in maintaining neuronal, haematopoietic and mammary gland stem cells. |
| SNAI1 | | Protein snai1 homolog 1 | Responsible for the degradation of E-cadherin and initiation of invasion |
| JAGGED2 | | Notch ligand Jagged | Stimulates Notch signaling |
| SFRP | | Secreted frizzled-related protein | Wnt antagonist |
| WNT | | Wnt family proteins or ligand | Representative of a WNT ligand |
| BCL2 | | Apoptosis regulator Bcl-2 | Anti-apoptotic |
| **V. Cellular Responses** | | | |
| Anti_Apop | | Anti apoptosis | These are the cellular responses or phenotypic expressions that have been shown as outcomes of this pathway. |
| Notch_Signal | | Nocth signaling |  |
| Wnt_Signal | | Wnt signaling |  |
| Cellcycle_Progression | | Cell cycle progression |  |
| Emt | | Epithelial to Mesenchymal transition |  |
| Cell_Proliferation | | Cellular proliferation |  |

* These proteins do not belong to the core proteins of Hedgehog pathway. We have considered these proteins so as to include direct cross talks by other molecules of different pathway which may influence different cancer scenarios.

In the above table, we have used the abbreviations of the proteins and cellular responses that are included in our reconstructed Hedgehog signaling pathway map. The entire table has been divided into five parts: I. Extracellular and Membrane Proteins. II. Cytoplasmic Proteins. III. Nuclear Proteins. IV. Output Proteins. V. Cellular Responses. The names of the proteins and corresponding documentation are taken from the databases listed in Table S1 and from the literatures [1-32].

**Table S4. Logical States of the Input and Output proteins of Hedgehog signaling in Normal, Glioma, Colon and Pancreatic Cancer Scenarios.**

| **Normal** | | | | | | **Glioma** | | | | **Colon** | | | | **Pancreatic** | | | |
| --- | --- | --- | --- | --- | --- | --- | --- | --- | --- | --- | --- | --- | --- | --- | --- | --- | --- |
| **Input** | | | **Output** | | | **Input** | | **Output** | | **Input** | | **Output** | | **Input** | | **Output** | |
| BMP_RUNX3 | | 0 | DHH | | 0 | SHH | 1 | DHH | 1 | SHH | 1 | DHH | 0 | DHH | 1 | DHH | 1 |
| DISPATCHED | | 1 | IHH | | 0 | DHH | 1 | IHH | 1 | IHH | 1 | SHH | 1 | SHH | 1 | SHH | 1 |
| HHAT | | 1 | SHH | | 1 | IHH | 1 | SHH | 1 | BMP_RUNX3 | 1 | IHH | 1 | IHH | 1 | IHH |  |
| CDO | | 1 | PTCH1 | | 1 | BMP_RUNX3 | 1 | PTCH1 | 0 | DISPATCHED | 1 | PTCH1 | 1 | BMP_RUNX3 | 1 | PTCH1 | 1 |
| BOC | | 1 | PTCH2 | | 0 | DISPATCHED | 1 | PTCH2 | 0 | HHAT | 1 | PTCH2 | 0 | DISPATCHED | 1 | PTCH2 | 0 |
| GAS1 | | 0 | SMO | | 1 | HHAT | 1 | SMO | 0 | CDO | 1 | SMO | 1 | HHAT | 1 | SMO | 1 |
| HFU | | 0 | STK36 | | 1 | CDO | 1 | STK36 | 0 | BOC | 1 | STK36 | 1 | CDO | 1 | STK36 | 1 |
| ULK3 | | 0 | GLI1 | | 1 | BOC | 1 | GLI1 | 0 | GAS1 | 0 | GLI1 | 1 | BOC | 1 | GLI1 | 1 |
| NOTCH1 | | 0 | GLI2 | | 1 | GAS1* | 0 | GLI2 | 0 | HFU | 1 | GLI2 | 1 | GAS1 | 0 | GLI2 | 1 |
| SUFU | | 0 | NUC_GLI1 | | 1 | GLI1 | 0 | NUC_GLI1 | 0 | ULK3 | 1 | NUC_GLI1 | 1 | HFU | 1 | NUC_GLI1 | 1 |
| TWIST | | 0 | NUC_GLI2 | | 1 | GLI2 | 0 | NUC_GLI2 | 0 | NOTCH1 | 0 | NUC_GLI2 | 1 | ULK3 | 1 | NUC_GLI2 | 1 |
| RAS | | 0 | GLI3_A | | 1 | HFU | 1 | GLI3_A | 0 | SUFU* | 0 | GLI3_A | 1 | NOTCH1 | 0 | GLLI3_A | 1 |
| ERK12 | | 0 | GLI3_R | | 0 | ULK3 | 1 | GLI3_R | 1 | TWIST | 0 | GLI3_R | 0 | SUFU* | 0 | GLI3_R | 0 |
| PKA_A | | 0 | FAS | | 0 | NOTCH1 | 0 | FAS | 1 | RAS | 1 | FAS | 0 | TWIST | 0 | FAS | 0 |
| BTRCP | | 0 | CYCLIN_B | | 1 | SUFU* | 0 | CYCLIN_B | 0 | ERK12 | 0 | CYCLIN_B | 1 | RAS | 1 | CYCLIN_B | 1 |
| CKI_A | | 0 | CYCLIN_D | | 1 | TWIST | 1 | CYCLIN_D | 0 | PKA_A* | 0 | CYCLIN_D | 1 | ERK12 | 1 | CYCLIN_D | 1 |
| GSK3 | | 0 | CYCLIN_D2 | | 1 | RAS | 1 | CYCLIN_D2 | 0 | BTRCP* | 0 | CYCLIN_D2 | 1 | PKA_A* | 0 | CYCLIN_D2 | 1 |
| DYRK1 | | 1 | CYCLIN_E | | 1 | ERK12 | 1 | CYCLIN_E | 0 | CKI_A* | 0 | CYCLIN_E | 1 | BTRCP* | 0 | CYCLIN_E | 1 |
| NUMB | | 0 | FOXM1 | | 1 | PKA_A* | 0 | FOXM1 | 0 | GSK3* | 0 | FOXM1 | 1 | CKI_A* | 0 | FOXM1 | 1 |
| ITCH | | 0 | PDGFRA | | 1 | BTRCP* | 0 | PDGFRA | 0 | DYRK1 | 1 | PDGFRA | 1 | GSK3* | 0 | PDGFRA | 1 |
| **Normal** | | | | | | **Glioma** | | | | **Colon** | | | | **Pancreatic** | | | |
| **Input** | | | **Output** | | | **Input** | | **Output** | | **Input** | | **Output** | | **Input** | | **Output** | |
| SKI | 0 | | CTNNB_TCF4 | 0 | | CKI_A* | 0 | CTNNB_TCF4 | 1 | NUMB* | 0 | CTNNB_TCF4 | 0 | DYRK1 | 1 | CTNNB_TCF4 | 0 |
| NCOR | 0 | | OPN | 1 | | GSK3* | 0 | OPN | 0 | ITCH* | 0 | OPN | 1 | NUMB* | 0 | OPN | 1 |
| HDAC | 0 | | CMYC | 1 | | DYRK1 | 1 | CMYC | 0 | SKI* | 0 | CMYC | 1 | ITCH* | 0 | CMYC | 1 |
| SNO | 0 | | BMI | 1 | | NUMB* | 0 | BMI | 0 | NCOR* | 0 | BMI | 1 | SKI* | 0 | BMI | 1 |
| SIN3A | 0 | | SNAI1 | 1 | | ITCH* | 0 | SNAI1 | 0 | HDAC* | 0 | SNAI1 | 1 | NCOR* | 0 | SNAI1 | 1 |
| NUC_STK36 | 1 | | JAGGED2 | 1 | | SKI* | 0 | JAGGED2 | 0 | SNO* | 0 | JAGGED2 | 1 | HDAC* | 0 | JAGGED2 | 1 |
| NUC_SUFU | 0 | | SFRP | 1 | | NCOR* | 0 | SFRP | 0 | SIN3A* | 0 | SFRP | 1 | SNO* | 0 | SFRP | 1 |
| NA | NA | | WNT | 1 | | HDAC* | 0 | WNT | 0 | NUC_STK36 | 1 | WNT | 1 | SIN3A* | 0 | WNT | 1 |
| NA | NA | | BCL2 | 1 | | SNO* | 0 | BCL2 | 0 | NUC_SUFU* | 0 | BCL2 | 1 | NUC_STK36 | 1 | BCL2 | 1 |
| NA | NA | | NA | NA | | SIN3A* | 0 | HHIP | 1 | NA | NA | HHIP | 1 | NUC_SUFU* | 0 | NA | NA |
| NA | NA | | NA | NA | | NUC_STK36 | 1 | NA | NA | NA | NA | NA | NA | NA | NA | NA | NA |
| NA | NA | | NA | NA | | NUC_SUFU* | 0 | NA | NA | NA | NA | NA | NA | NA | NA | NA | NA |

NA: Not Applicable

* Proteins having Loss of Function in cancer scenario.

This table shows the Logical states used in CellNetAnalyzer for simulating the Hedgehog Pathway model of Normal, Glioma, Colon and Pancreatic cancer scenarios. The logical states of the input proteins for each scenario and the respective simulated results of the output proteins are given in this table. The logical state ‘1’of a node represents its expression or as "ON" state where "0" represents the "OFF" state. The Logical states of the input proteins have considered from various database (See Table S1) and literature sources [1-3,9,10,18,22,26,32,33,35,36-62 ]. Here we have only shown the simulation results of "Time scale 2" for each scenario.

**Formulation of Logical equations:**

In Table S5, we have shown all the logical or Boolean equations which were used to simulate the pathway in CellNetAnalyzer. In the second column, documentation of the equations with references has been given. As an example, in order to release the active hedgehog ligand ‘Sonic Hedgehog (SHH)’ from a transducer cell to effectors cell, both Dispatched as well as Hedgehog acyltransferase (HHAT) are required for its release and maturation. Thus, the matured and active state of SHH depends on the active states of both Dispatched and HHAT. Therefore in logical term the output of SHH is regulated by Dispathced and HHAT proteins. So this interaction can be represented as:

**DISPATCHED AND HHAT 🡺 SHH (General Boolean notation)**

**or,**

**DISPATCHED + HHAT 🡺 SHH (CNA notation)**

The ‘NOT’ logical operator can also be used with the ‘AND’ logical operator to depict that the state of a species depends on the active form of a species ‘A’ and inactive form of a species ‘B’, i.e. it is subject to the activating and inhibitory influence of both the species. For example, in the absence of a hedgehog signal, the Patched1/2 (Ptch1/2) receptor suppresses the activity of Smoothened (SMO) protein in the membrane. Upon binding of the hedgehog ligands to the Patched receptor, this inhibition is been removed and Smoothened (SMO) will be active. Thus the presence of the hedgehog ligands and the absence of the inhibitory effect of the Patched receptor enable the activation of SMO which can be represented as:

**SHH AND NOT PTCH1_Free 🡺 SMO (General Boolean notation)**

**or,**

**SHH +! PTCH1_Free = SMO (CNA notation)**

The ‘OR’ logical operator enables the representation of interactions which activate/inactivate a particular species independent of each other. Thus the two events are mutually exclusive of each other. For example, GLI1 (transcription factor) is activated by several modes such as via GLI2, TWIST [13], [14]. Thus the state of GLI1 (0 or 1) depends on either GLI2 or TWIST. These interactions can be represented as:

**GLI2 OR TWIST🡺 Gli1 (General Boolean notation)**

**or,**

**GLI2 = GLI1 (CNA notation)**

**TWIST = GLI1**

In our model, we have also incorporated early as well as late events which are responsible for the determination of the state of a species. This modeling is necessary to resolve issues in case of that interaction where a particular reaction becomes active significantly later than the other. In order to model such events, we have assigned different time scale value to those interactions. As an example, it is proved that after the activation of GLI1 protein in the cytoplasm, it come into the nucleus as NUC_GLI1and activates the transcription of various target genes of Hedgehog pathway. Now, to simulate these procedures in our Boolean model, we have divided these whole procedures in two different time scales. At time scale 1, we have considered the accumulation of active GLI proteins in the nucleus from cytoplasm and at time scale 2, the activation of the Hedgehog target gens or the output proteins by the nuclear GLI proteins and several other nuclear transcription factors have been considered. Therefore, in terms of Boolean interactions our model was:

**GLI1= NUC_GLI1 (Time Scale: 1)**

**NUC_GLI1+NUC_STK36+DYRK1+! NUC_SUFU+!NUMB+!ITCH = GLI1(Time Scale: 2)**

**Table S5. Boolean equations with the documentation for the Hedgehog pathway.**

| **Interactions** | **Documentation** |
| --- | --- |
| 🡺DISPATCHED | Inputs to the model. Upstream regulators of these molecules have not been considered. |
| 🡺HHAT |  |
| 🡺 CDO |  |
| 🡺 BOC |  |
| 🡺 NUC_SUFU |  |
| 🡺 GAS1 |  |
| 🡺BMP_RUNX3 |  |
| 🡺ULK3 |  |
| 🡺 HFU |  |
| 🡺 SUFU |  |
| 🡺 ERK12 |  |
| 🡺 RAS |  |
| 🡺 TWIST |  |
| 🡺 DYRK1 |  |
| 🡺 NUMB |  |
| 🡺 ITCH |  |
| 🡺 PKA_ALPHA |  |
| 🡺 BTRCP |  |
| 🡺 CKI_A |  |
| 🡺 GSK3 |  |
| 🡺 NUC_STK36 | Inputs to the model. Upstream regulators of these molecules have not been considered. |
| 🡺 NOTCH1 |  |
| 🡺 SKI |  |
| 🡺 SNO |  |
| 🡺 NCOR |  |
| 🡺 SIN3 ALPHA |  |
| 🡺 HDAC |  |
| BMP_RUNX3🡺IHH | BMP-RUNX3 signaling induces expression of IHH in surface differentiated epithelial cells of stomach and intestine [1]. |
| CDO+BOC🡺SHH | CDO and BOC bind SHH through a high-affinity interaction with a speciﬁc ﬁbronectin repeat that is essential for activity. They demonstrate that CDO and BOC are necessary but not sufficient for activation [2]. However, there is no evidence for the exact mechanism and if both are required for the enhancement of signaling. |
| DISPATCHED+HHAT+!HHIP🡺DHH | Dispatched regulates the release and extracellular accumulation of cholesterol-modified hedgehog proteins and is hence required for effective production of the HH signal [3], [4]. HHAT (Hedgehog acyltransferase) is a hedgehog modifier which induces lipid modification to generate mature peptides. HH proteins with lipid modification are then released from producing cells by Dispatched homologues [1]. HHIP can antagonize all types of HH ligands [5]. |
| DISPATCHED+HHAT+!HHIP🡺IHH | Dispatched and HHAT system also operates in the same way as during DHH release [3], [4]. HHIP can antagonize all types of HH ligands [5]. |
| DISPATCHED+HHAT+!HHIP+!GAS1🡺SHH | Dispatched and HHAT also operate in the same way as during DHH release [3], [4]. HHIP is found to bind directly to SHH and attenuate SHH signaling like PTCH1/2 while its expression was induced by SHH signals [5], [6]. |
| !DHH+!IHH+!SHH🡺PTCH1_Free | Negative influence of all the Hedgehog ligands was considered to denote the inactive state of Patched (PTCH1 and PTCH2) receptors. In the absence of Hedgehog ligands Patched receptors are active and suppress the activity of Smoothened [7]. |
| !DHH+!IHH+!SHH🡺PTCH2_Free |  |
| DHH+!PTCH1_Free🡺SMO | In the absence of a stimulus by Hedgehog, Patched receptor inhibits Smoothened. Upon binding of Hedgehog ligands DHH, SHH or IHH to Patched , Smoothened is activated leading to the transcription of target genes. This is also reported that mutations affecting the transmembrane proteins Patched or Smoothened trigger the ligand independent activity of Hedgehog signaling pathway and are hence associated with human tumors such as basal cell carcinoma and medulloblastoma [7]. |
| IHH+!PTCH1_Free🡺SMO |  |
| SHH+!PTCH1_Free🡺SMO |  |
| SHH+!PTCH2_Free🡺SMO |  |
| SMO🡺STK36 | SMO binds to STK36 to stabilize GLI proteins [1]. |
| !SMO🡺FAS | SMO expression inhibits FAS thereby preventing apoptosis [8]. |
| HFU+!PKA_A+!GSK3+!CKI_A+!BTRCP+!SUFU🡺GLI1 | HFU enhances GLI1 function in a manner that is independent of a functional kinase domain [9]. GSK3 phosphorylates GLI proteins post phosphorylation by PKA and it is known to elicit negative effects [10]. SMO inactivation leads to formation of the cytoplasmic GLI degradation complex, in which GLI family members (GLI1, GLI2 and GLI3) are phosphorylated by casein kinase alpha (CKI_α), glycogen synthase kinase-3ß (GSK3ß) and protein kinase A (PKA). Phosphorylated GLI is recognized by FBXW1/BTRCP1 and FBXW11/BTRCP2 for ubiquitination, and ubiquitinated GLI is partially degraded to release its intact N-terminal half thereby functioning as transcriptional repressor [1]. The inhibitory interactions have been included with activation interactions using an AND operator. Therefore GLI cannot be activated unless and until all the inhibitors are absent. However, this needs to be checked in *in vivo* conditions. But in the model it is necessary to introduce these interactions using an "AND" operation to assure signal flow. |
| ERK12+!PKA_A+!GSK3+!CKI_A+!BTRCP+!SUFU🡺GLI1 | EGFR signals via ERK potentiate target gene activation via GLI1 [11]. |
| RAS+!PKA_A+!GSK3+!CKI_A+!BTRCP+!SUFU🡺GLI1 | It is reported that oncogenic KRAS/ constitutively active RAS in Pancreatic Cancer cells, increases the transcription of GLI1 levels [12]. |
| TWIST+!PKA_A+!GSK3+!CKI_A+!BTRCP+!SUFU🡺GLI1 | TWIST activates human GLI1 regulatory sequences via two E-boxes in GLI1's first intron. Demonstrated in a murine model and using human GLI sequences. Two critical cis elements in human GLI1 gene: a GC box that binds Sp1 at 195 and two E-boxes that operate at 157 and 482 have also identified. The 157 E-box binds USF1 and USF2, while E-box 482 binds TWIST. Sp1 and USf1/2 are ubiquitiously expressed TFs and can function either as activators or repressors depending upon cellular context. However their roles have not been clearly delineated and hence not incorporated in the model [13]. |
| ULK3+!PKA_A+!GSK3+!CKI_A+!BTRCP+!SUFU🡺GLI1 | ULK3, a Ser/Threonine kinase present in addition to STK36 is essential for the up-regulation of GLI transcriptional activity. It phosphorylates GLI1 in both N (1-426) and C (754 -1126) terminus but the fragment of gli1 between residues 426 -754 is not phosphorylated by ULK3. Thus ULK3 is a positive activator [10]. |
| STK36+!PKA_A+!GSK3+!CKI_A+!BTRCP+!SUFU🡺GLI1 | STK36 is a positive regulator of SHH pathway that acts independent on is functional kinase domain. STK36 enhances GLI2 activity but not GLI1 in C3H10T1/2 and HEK293 cells and Gli1 transcriptional activity in NIH3T3C2. Sn480 cells. Hence STK36 expression is cell type specific. As ours is a master model we have nevertheless included this interaction [10]. |
| GLI2+!PKA_A+!GSK3+!CKI_A+!BTRCP+!SUFU🡺GLI1 | GLI1 is a direct target of GLI2. The study was conducted in normal human epidermis and Basal cell carcinoma cells [14]. |
| GLI3_A+!SKI+!SNO+!NCOR+!SIN3A+!HDAC🡺GLI1 | GLI3 exists in two forms — a full-length transcriptional activator (GLI3A) or an amino-terminal fragment that functions as a repressor. This particular activator isoform is GLI3A [15]. On the other hand SKI, SKI related protein SNO, NCOR, SIN3A, HDACs form a transcriptional repressor complex that interfere with GLI1 activation by full length GLI3 [16]. Hence they are included in the AND interaction. |
| GLI1🡺FOXM1 | FOXM1/FOXL1 is a direct target of GLI mediated activation [1]. |
| FOXM1🡺CELL_PROLIFERATION | FOXM1 is known to mediate cell proliferative functions [17]. |
| CELL_PROLIFERATION🡺 | Cellular response in our model. |
| !GLI1🡺GLI3_R | Repressor form of GLI3 produced in the absence of GLI1 expression [18]. |
| ! GLI3_R 🡺 GLI3_ACTIVE | GLI3_ACTIVE form is produced in the absence of GLI3 repressor form [18] |
| GLI1🡺PDGFRA | PDGFRA is expressed at high levels in human and murine Basal Cell Carcinoma. It has been found that ectopic expression of GLI increases PDGFRA levels i.e. increases receptor protein levels whereas inhibition of the HH pathway reduces PDGFRA levels [19]. |
| PDGFRA🡺 |  |
| GLI1🡺NUC_GLI1 | Cellular location of GLI1 has been found in Cytoplasm as well as in Nucleus [3]. We considered this transportation in our model and named NUC_GLI1 of the nuclear counterpart of GLI1. |
| NUC_GLI1+NUC_STK36+DYRK1+!NUC_SUFU+!NUMB+!ITCH🡺PTCH1 | PTCH 1 and HHIP receptors inhibit the pathway in the absence of a stimulus or Hedgehog ligands. This indicates the formation of a negative feedback loop [19]. DYRK1 (Dual Specificity Tyrosine phosphorylation Regulated Kinase 1A / Dual Specificity Yak1 related Kinase) can substantially enhance GLI1 dependent transcription. It has also been suggested that failure of SHH to stimulate DYRK1 kinase activity is indicative to the fact that DYRK1 may not be regulated by the SHH signaling pathway but functionally interacts with it [20]. SUFU inhibits the activator isoform of GLI proteins and activates the repressor forms [21]. A proper balance between both the forms regulates Wnt signaling. It is also reported that NUMB along with ubiquitin ligase such as ITCH is able to polyubiquitinate Gli1 and target it for degradation and thus control HH signaling. Thus all these components have been included in the AND equation as they affect the final outcome. |
| NUC_GLI1+NUC_STK36+DYRK1+!NUC_SUFU+!NUMB+!ITCH🡺HHIP |  |
| NUC_GLI1+NUC_STK36+DYRK1+!NUC_SUFU+!NUMB+!ITCH🡺GLI1 | It is reported GLI1 is also produced at the end of this pathway and thus create a positive feedback loop [22], [23]. |
| NUC_GLI1+NUC_STK36+DYRK1+!NUC_SUFU+!NUMB+!ITCH🡺OPN | Osteopontin (OPN) is a direct transcriptional target of GLI1demonstrated in MDA-MB 435 cell line. OPN is a secreted protein that influences multiple downstream signaling events that allow cancer cells to resist apoptosis, invade through ECM, evade host immunity and influence growth of indolent tumors. OPN is expressed by normal cells. However sustained expression in cancer cells promotes aberrant growth of cells and an invasive phenotype [24]. |
| OPN 🡺 |  |
| NUC_GLI1+NUC_STK36+DYRK1+!NUC_SUFU+!NUMB+!ITCH🡺CYCLIN_D | Hedgehog signaling regulates the proliferation of distinct cell types via direct activation of genes that are involved in cell cycle progression and mediate G1 to S transition. |
| NUC_GLI1+NUC_STK36+DYRK1+!NUC_SUFU+!NUMB+!ITCH🡺CYCLIN_E |  |
| CYCLIN_D🡺CELLCYCLE_PROGRESSION | Cyclins such as CYCLIN D and CYCLIN E are involved in regulation of cell cycle. |
| CYCLIN_E🡺CELLCYCLE_PROGRESSION |  |
| CELLCYCLE_PROGRESSION🡺 | Cell cycle progression is one of the cellular responses that have been considered in our model. |
| NUC_GLI1+NUC_STK36+DYRK1+!NUC_SUFU+!NUMB+!ITCH🡺CMYC | Expression of GLI1 and C-MYC has also found in various experiments [25], [26]. |
| CMYC🡺 |  |
| NUC_GLI1+NUC_STK36+DYRK1+!NUC_SUFU+!NUMB+!ITCH🡺BMI | Hedgehog signalling leads to an increased expression of BMI-1 in isolated mammary epithelial stem cells and CSCs. BMI-1 is a transcriptional repressor belonging to the polycomb gene family and its suppressor functions are involved in maintaining neuronal, haematopoietic and mammary gland stem cells. It leads to self renewal [11]. Activated STK36 also phosphorylates SUFU to promote the nuclear accumulation of full length GLI [1]. It is also reported that activation of hedgehog signaling increases mammosphere- initiating cell number and mammosphere size, whereas inhibition of the pathway results in a reduction of these effects. These effects are mediated by the polycomb gene *bmi* [27]. |
| BMI🡺 | Output protein considered in our model. |
| NUC_GLI1+NUC_STK36+DYRK1+!NUC_SUFU+!NUMB+!ITCH🡺SNAI1 | It is reported that activation of SNAI1 a protein responsible for degradation of cadherin and induction of invasion is directly activated by GLI1 [11]. SNAI1 protein is responsible for epithelial to mesenchymal transition. |
| SNAI1🡺EMT |  |
| EMT🡺 | Cellular response Epithelial to Mesenchymal Transition. |
| NUC_GLI1+NUC_STK36+DYRK1+!NUC_SUFU+!NUMB+!ITCH🡺JAGGED2 | Expression of JAGGED2 has been reported [22]. |
| JAGGED2🡺NOTCH_SIGNAL | JAGGED2 is a notch ligand, hence promotes notch signaling. |
| NOTCH_SIGNAL🡺 | Cellular response. |
| NUC_GLI1+NUC_STK36+DYRK1+!NUC_SUFU+!NUMB+!ITCH🡺SFRP | Expression of SFRP by GLI1 has been found [22], [28]. |
| NUC_GLI1+NUC_STK36+DYRK1+!NUC_SUFU+!NUMB+!ITCH🡺WNT | GLI1 mediates the activation of wnt family proteins and enhances signaling via these pathways, exact wnt ligand is not known and thus not included in our model [18]. |
| WNT+!SFRP🡺WNT_SIGNAL | Activation of Wnt signaling depends on the presence of WNT ligand and absence of its antagonist SFRP [29]. |
| WNT_SIGNAL🡺 | Cellular response. |
| NUC_GLI1+NUC_STK36+DYRK1+!NUC_SUFU+!NUMB+!ITCH🡺BCL2 | In epidermal cells GLI1 can induce the expression of antiapoptotic factor BCL2 [21]. |
| HFU+!PKA_A+!GSK3+!CKI_A+!BTRCP+!SUFU🡺GLI2 | HFU enhances GLI2 function in a manner that is independent of a functional kinase domain [9]. |
| STK36+!PKA_A+!GSK3+!CKI_A+!BTRCP+!SUFU🡺GLI2 | STK36 is a positive regulator of GLI2 activity [10]. STK36 enhances GLI2 activity but not GLI1 in C3H10T1/2 and HEK293 cells. GSK3 phosphorylates GLI proteins post phosphorylation by PKA and it is known to elicit negative effects. On the other hand SMO inactivation leads to formation of the cytoplasmic GLI degradation complex, in which GLI family members (GLI1, GLI2 and GLI3) are phosphorylated by casein kinase I (CKI), glycogen synthase kinase-3ß (GSK3ß) and protein kinase A (PKA) [1]. Phosphorylated GLI is recognized by FBXW1/BTRCP1 and FBXW11/BTRCP2 for ubiquitination, and ubiquitinated GLI is partially degraded to release its intact N-terminal half functioning as transcriptional repressor. Thus all the above factors are included in the AND equation as they influence the formation of GLI2 |
| HFU+!NOTCH1🡺GLI2 | HFU enhances GLI2 function in a manner that is independent of a functional kinase domain [9]. Inactivation of notch1 gene in epidermis induces sustained expression of GLI2 and causes Basal Cell Carcinoma [19]. Thus, GLI2 is activated in the presence of HFU and absence of notch1. This interaction has been represented as an AND equation. |
| STK36+!NOTCH1🡺GLI2 | We have represented an alternative mode of GLI2 activation by STK36 without the presence of NOTCH1. |
| GLI2🡺NUC_GLI2 | GLI2 when transported to the nucleus is represented as NUC_GLI2. |
| NUC_GLI2+NUC_STK36+!NUC_SUFU🡺CYCLIN_D2 | However, this interaction has been found in a murine model [21], [30], we included this interaction as CYCLIN_D2 is one of the important proteins for cellcycle progression. Activated STK36 also phosphorylates SUFU to promote the nuclear accumulation of full length GLI. |
| CYCLIN_D2🡺CELLCYCLE_PROGRESSION | CYCLIN D2 is implicated in cell cycle regulation. |
| NUC_GLI2+NUC_STK36+!NUC_SUFU🡺BCL2 | Epidermal cells GLI2 can induce the expression of anti apoptotic factor BCL2 [21]. |
| BCL2🡺ANTI_APOP | BCL2 is a known anti-apoptotic factor [31]. |
| ANTI_APOP🡺 | Cellular responses. |
| !GLI1 🡺 CTNNB_TCF4 | Coincident of high-to-low TCF and low-to-high HH-GLI1 transitions in patient Colon Carcinoma have been found. Therefore, we can write that higher level of expression of GLI1 inhibits the activity of TCF complex [62]. |
| CTNNB_TCF4 🡺 |  |
| GLI3_A+NUC_STK36+!NUC_SUFU🡺CYCLIN_D2 | Similarly active form of GLI3 mediates the activation of CYCLIN_D2. |
| !PTCH1🡺CYCLIN_B | It is reported that PTCH regulates the activity of CYCLIN_B. Interaction with Patched in the cytoplasm blocks cell proliferation by preventing nuclear localization of the activated complex. Ligand induced activation of this complex leads to the nuclear localization of CYCLIN B by disruption of the physical interaction between Patched 1 and CYCLIN B [19]. |
| CYCLIN_B🡺CELLCYCLE_PROGRESSION | CYCLIN B is also implicated in cell cycle regulation. |

**Table S6. Comparison of the percentage of accuracy between experimental and simulation results for each cancer scenario.**

| **Disease** | **Comparison** | **Not Determined/ Not Available** | **Correct Predictions** | **Incorrect Predictions** | ***Accuracy (in %)** |
| --- | --- | --- | --- | --- | --- |
| **Glioma** | **SIM1 with EXP** | **24** | **22** | **11** | **66.66** |
|  | **SIM2 with SIM1** | **0** | **54** | **3** | **94.37** |
|  | **SIM2 with EXP** | **24** | **25** | **8** | **75.75** |
| **Colon Cancer** | **SIM1 with EXP** | **52** | **5** | **0** | **100.00** |
|  | **SIM2 with SIM1** | **0** | **57** | **57** | **100.00** |
|  | **SIM2 with EXP** | **52** | **5** | **0** | **100.00** |
| **Pancreatic Cancer** | **SIM1 with EXP** | **13** | **25** | **19** | **56.80** |
|  | **SIM2 with SIM1** | **0** | **47** | **10** | **82.45** |
|  | **SIM2 with EXP** | **13** | **32** | **12** | **72.72** |

***Accuracy (%) = ((Correct Predictions) / (Correct Predictions) + (Incorrect Predictions)) X 100 (%)**

**References:**

1. Katoh Y., Katoh M. (2006) Hedgehog signaling pathway and gastrointestinal stem cell signaling network. Int J Mol Med 18(6):1019-1023.

2. Tenzen T., Allen B. L., Cole F., Kang J.S., Krauss R. S., McMahon A. P. (2006) The cell surface membrane proteins Cdo and Boc are components and targets of the Hedgehog signaling pathway and feedback network in mice. Dev Cell 10(5):647-656. doi: 10.1016/j.devcel.2006.04.004.

3. The UniProt Consortium (2012) Reorganizing the protein space at the Universal Protein Resource (UniProt). Nucleic Acids Res. 40: D71-D75.

4. Evangelista M, Tian H, de Sauvage FJ (2006) The hedgehog signaling pathway in cancer. Clin. Cancer Res. 12(20 Pt 1):5924-59288. doi: 10.1158/1078-0432.CCR-06-1736.

5. Olsen CL, Hsu PP, Glienke J, Rubanyi GM, Brooks AR (2004) Hedgehog-interacting protein is highly expressed in endothelial cells but down-regulated during angiogenesis and in several human tumors. BMC Cancer 4:43: 1471-2407. doi: 10.1186/1471-2407-4-43.

6. Tojo M, Kiyosawa H, Iwatsuki K, Kaneko F (2002) Expression of a sonic hedgehog signal transducer , hedgehog-interacting protein , by human basal cell carcinoma. British Journal of Dermatology 146(1):69-73. doi: 10.1046/j.1365-2133.2002.04583.x.

7. Taipale J, Cooper MK, Maiti T, Beachy PA (2002) Patched acts catalytically to suppress the activity of Smoothened. Nature 418:892-896. doi:10.1038/nature00989.

8. Athar M, Li C, Tang X, Chi S, Zhang X et al. (2004) Inhibition of smoothened signaling prevents ultraviolet B-induced basal cell carcinomas through regulation of Fas expression and apoptosis. Cancer Res 64:7545-7552 doi:10.1158/0008-5472.CAN-04-1393.

9. Østerlund T, Kogerman P (2006) Hedgehog signalling: how to get from Smo to Ci and Gli. Trends in Cell Biology 16(4):176-180. doi: 10.1016/j.tcb.2006.02.004.

10. Maloverjan A, Piirsoo M, Michelson P, Kogerman P, Østerlund T (2009) Identification of a novel serine / threonine kinase ULK3 as a positive regulator of Hedgehog pathway. Exp Cell Res 316(4):627-637. http://dx.doi.org/10.1016/j.yexcr.2009.10.018.

11. Kasper M, Jaks V, Fiaschi M, Toftgård R (2009) Hedgehog signalling in breast cancer. Carcinogenesis, 30(6):903-11. doi: 10.1093/carcin/bgp048.

12. Ji Z, Mei FC, Xie J, Cheng X (2007) Oncogenic KRAS activates hedgehog signaling pathway in pancreatic cancer cells. Jour Biol Chem 282(19):14048-55. doi: 10.1074/jbc.M611089200.

13. Villavicencio EH, Yoon JW, Frank DJ, Füchtbauer EM, Walterhouse DO et al.,(2002) Cooperative E-Box Regulation of Human GLI1 by TWIST and USF. Genesis 32(4):247-258. doi: 10.1002/gene.10078.

14. Ikram MS, Neill GW, Regl G, Eichberger T, Frischauf AM et al. (2004) GLI2 is expressed in normal human epidermis and BCC and induces GLI1 expression by binding to its promoter. Journal of Investigative Dermatology;122(6):1503-9.

15. Rohatgi R, Scott MP (2007) Patching the gaps in Hedgehog signalling. Nat Cell Biol 9(9):1005-1009. doi: 10.1038/ncb435.

16. Dai P, Shinagawa T, Nomura T (2002) Ski is involved in transcriptional regulation by the repressor and full-length forms of Gli3. Genes & Dev.16:2843-2848. doi:10.1101/gad.1017302.

17. Gong A, Huang S (2012) FoxM1 and Wnt/β-Catenin Signaling in Glioma Stem Cells. Cancer Res.;72(22):5658-62.

18. Ruiz I Altaba A, Sánchez P, Dahmane N (2002) Gli and hedgehog in cancer: tumours, embryos and stem cells. Nat Rev Cancer 2(5):361-72. doi: 10.1038/nrc796.

19. Pasca MM, Hebrok M (2003) Hedgehog signalling in cancer formation and maintenance. Nat Rev Cancer 3(12):903-911. doi:10.1038/nrc1229.

20. Mao J, Maye P, Kogerman P, Tejedor FJ, Toftgard R et al. (2002) Regulation of Gli1 Transcriptional Activity in the Nucleus by Dyrk1. J Biol Chem 277(38):35156 -35161. doi:10.1074/jbc.M206743200.

21. Kasper M, Jaks V, Fiaschi M, Toftgård R (2009) Hedgehog signalling in breast cancer. Carcinogenesis, 30(6):903-11. doi: 10.1093/carcin/bgp048.

22. Katoh M (2007) Networking of WNT , FGF , Notch , BMP , and Hedgehog Signaling Pathways during Carcinogenesis. Stem Cell Reviews and Reports. 3(1):30-38. doi: 10.1007/s12015-007-0006-6.

23. Jason K.Sicklick, Yin-Xiong Li, Aruna Jayaraman, Rajesh Kannangai, Yi Qi, Perumal Vivekanandan et al. (2006) Dysregulation of the Hedgehog pathway in human hepatocarcinogenesis. Carcinogenesis vol.27 no.4 pp.748–757.

24. Das S, Harris LG, Metge BJ, Liu S, Riker AI, et al. (2009) The hedgehog pathway transcription factor GLI1 promotes malignant behavior of cancer cells by up-regulating osteopontin. J. Biol. Chem., 284(34), 22888-97. doi: 10.1074/jbc.M109.021949.

25. Iu´ri D. Louro, Evans C. Bailey, Xingnan Li, Lindsey S. South, Peggy R. McKie-Bell et al. (2002) Comparative Gene Expression Profile Analysis of GLI and c-MYC in an Epithelial Model of Malignant Transformation. CANCER RESEARCH; 62:5867–5873

26. Sandra H. Bigner,Henry S. Friedman, Bert Vogelstein, VV.Jerry Oakes, Darell D. Bigner (1990)Amplification of the c-myc Gene in Human Medulloblastoma Cell Lines and Xenografts Cancer Research; 50:2347-2350.

27. Liu S, Dontu G, Mantle ID, Patel S, Ahn, N.-shik, Jackson, K. W. et al. (2006) Hedgehog signaling and Bmi-1 regulate self-renewal of normal and malignant human mammary stem cells. Cancer Res; 66(12):6063-71. doi: 10.1158/0008-5472.CAN-06-0054.

28. He J, Sheng T, Stelter AA, Li C, Zhang X, Sinha M, et al. (2006) Suppressing Wnt signaling by the hedgehog pathway through sFRP-1. Jour Biol Chem 281(47):35598-35602. doi: 10.1074/jbc.C600200200.

29. David J. Elzi, Meihua Song, Kevin Hakala, Susan T. Weintraub, Yuzuru Shiio (2012) Wnt antagonist SFRP1 functions as secreted mediator of senescence. Mol. Cell. Biol. MCB.06023-11.

30. Buttitta L, Mo R, Hui CC and Fan CM (2003). Interplays of Gli2 and Gli3 and their requirement in mediating Shh-dependent sclerotome induction. Development (Cambridge, England), 130(25):6233-43. doi: 10.1242/dev.00851.

31. Atan Gross, James M. McDonnell,Stanley J. Korsmeyer (1999) BCL-2 family members and the mitochondria in apoptosis. Genes & Dev.13: 1899-1911.

32. Lawrence Lum, Chi Zhang, Sekyung Oh,Randall K. Mann, Doris P. von Kessler et al. (2003) Hedgehog Signal Transduction via Smoothened Association with a Cytoplasmic Complex Scaffolded by the Atypical Kinesin, Costal-2. Molecular Cell; 12:1261–1274.

33. Susanne Kaesler, Bernhard Lüscher, Ulrich Rüther (2005) Transcriptional Activity of GLI1 Is Negatively Regulated by Protein Kinase A. Biological Chemistry, Volume 381, Issue 7, Pages 545–551, DOI: 10.1515/BC.2000.070

34. Yuriko Katoh, Masaru Katoh (2005) Hedgehog Signaling Pathway and Gastric Cancer. Cancer Biology & Therapy 4:10, 1050-1054.

35. Lucia Di Marcotullio, Elisabetta Ferretti, Azzura Greco, Enrico De Smaele, Agnese Po et al. (2006) Numb is a suppressor of Hedgehog signalling and targets Gli1 for Itch-dependent ubiquitination. Nature Cell Biology 8, 1415 - 1423, DOI:10.1038/ncb1510.

36. Virginie Clement, Pilar Sanchez, Nicolas de Tribolet, Ivan Radovanovic, Ariel Ruiz i Altaba (2007) Hedgehog-Gli1 Signaling Regulates Human Glioma Growth, Cancer Stem Cell Self Renewal, and Tumorigenicity. Current Biology 17, 165–172. DOI 10.1016/j.cub.2006.11.033.

37. Eli E. Bar, Aneeka Chaudhry, Alex Lin, Xing Fan, Karisa Schreck et al. (2007) Cyclopamine-Mediated Hedgehog Pathway Inhibition Depletes Stem-Like Cancer Cells in Glioblastoma. Stem Cells, Volume 25, Issue 10, pages 2524–2533, DOI: 10.1634/stemcells.2007-0166.

38. Antony Hsieh, Ron Ellsworth, David Hsieh (2011) Hedgehog/GLI1 regulates IGF dependent malignant behaviors in glioma stem cells. Journal of Cellular Physiology, Volume 226, Issue 4, pages 1118–1127, DOI: 10.1002/jcp.22433.

39. Maria C Elias, Kathleen R Tozer, John R Silber, Svetlana Mikheeva, Mei Deng et al.(2005) TWIST is Expressed in Human Gliomas and Promotes Invasion. Neoplasia, 7(9): 824–837.

40. Hao Ding, Luba Roncari, Patrick Shannon, Xiaoli Wu, Nelson Lau et al. (2001)Astrocyte-specific Expression of Activated p21-ras Results in Malignant Astrocytoma Formation in a Transgenic Mouse Model of Human Gliomas. Cancer Res; 61: 3826-3836.

41. Hui-Wen Lo, Hu Zhu, Xinyu Cao, Amy Aldrich, Francis Ali-Osman (2009) A Novel Splice Variant of GLI1 That Promotes Glioblastoma Cell Migration and Invasion. Cancer Res, 69; 6790, DOI: 10.1158/0008-5472.CAN-09-0886.

42. Mingguang Liu, Bingbing Dai, Shin-Hyuk Kang, Kechen Ban, Feng-Ju Huang et al. (2006) FoxM1B Is Overexpressed in Human Glioblastomas and Critically Regulates the Tumorigenicity of Glioma Cells. Cancer Res; 66:3593-3602.

43. Heikki Joensuu, Marjut Puputti, Harri Sihto, Olli Tynninen, Nina N Nupponen (2005) Amplification of genes encoding KIT, PDGFRα and VEGFR2 receptor tyrosine kinases is frequent in glioblastoma multiforme. The Journal of Pathology, DOI: 10.1002/path.1823.

44. Saitoh Y, Kuratsu J, Takeshima H, Yamamoto S, Ushio Y (1995) Expression of osteopontin in human glioma. Its correlation with the malignancy. Lab Invest;72(1):55-63.

45. Büschges R, Weber RG, Actor B, Lichter P, Collins VP, Reifenberger G (1999) Amplification and expression of cyclin D genes (CCND1, CCND2 and CCND3) in human malignant gliomas. Brain Pathology, 9(3):435-42.

46. Xiang Zhang, Ming Zha, An-yang Huang, Zhou Fei, Wei Zhang, Xi-ling Wang (2005) The effect of cyclinD expression on cell proliferation in human gliomas. Journal of Clinical Neuroscience, Volume 12, Issue 2, Pages 166–168.

47. Arnab Chakravarti, Meaghan A. Delaney, Elizabeth Noll, Peter McL. Black, Jay S. Loeffler et al. (2001) Prognostic and Pathologic Significance of Quantitative Protein Expression Profiling in Human Gliomas. Clin Cancer Res; 7:2387-2395.

48. Jakub Godlewski, Michal O. Nowicki, Agnieszka Bronisz, Shanté Williams, Akihiro Otsuki et al. (2008) Targeting of the Bmi-1 Oncogene/Stem Cell Renewal Factor by MicroRNA-128 Inhibits Glioma Proliferation and Self-Renewal. Cancer Res; DOI: 10.1158/0008-5472.CAN-08-2629.

49. Sung-Pil Han, Ji-Hoon Kim, Myoung-Eun Han, Hey-Eun Sim, Ki-Sun Kim (2011) SNAI1 is Involved in the Proliferation and Migration of Glioblastoma Cells. Cell Mol Neurobiol ; 31:489–496. DOI 10.1007/s10571-010-9643-4.

50. Sandra Reichratha, Cornelia S.L. Müllerb, Beate Gleissnera, Michael Pfreundschuha, Thomas Vogt (2010) Notch- and vitamin D signaling in 1,25(OH)2D3-resistant glioblastoma multiforme (GBM) cell lines. The Journal of Steroid Biochemistry and Molecular Biology; Volume 121, Issues 1–2, Pages 420–424.

51. Roth W, Wild-Bode C, Platten M, Grimmel C, Melkonyan HS (2000) Secreted Frizzled-related proteins inhibit motility and promote growth of human malignant glioma cells. Oncogene,19(37):4210-20.

52. Douard R, Moutereau S, Pernet P, Chimingqi M, Allory Y et.al. (2006) Sonic Hedgehog–dependent proliferation in a series of patients with colorectal cancer. Surgery; 139(5):665-670.

53. Fre´de´ric Varnat, Arnaud Duquet, Monica Malerba, Marie Zbinden, Christophe Mas et al. (2009) Human colon cancer epithelial cells harbour active HEDGEHOG-GLI signalling that is essential for tumour growth, recurrence, metastasis and stem cell survival and expansion. EMBO Molecular Medicine, Volume 1, Issue 6-7, DOI 10.1002/emmm.200900039.

54. Lian Fan, Carmen V. Pepicelli, Christian C. Dibble, Winnie Catbagan, Jodi L. Zarycki (2004) Hedgehog Signaling Promotes Prostate Xenograft Tumor Growth. Endocrinology 145(8):3961–3970, DOI: 10.1210/en.2004-0079.

55. Atsuko Shimoyama, Masahiro Wada, Fumiyo Ikeda, Kenji Hata, Takuma Matsubara et al.(2007) Ihh/Gli2 Signaling Promotes Osteoblast Differentiation by Regulating Runx2 Expression and Function. Molecular Biology of the Cell, Vol. 18, 2411–2418.

56. Dr. Matsuhei Tanaka, Kenji Omura,Yoh Watanabe,Yoshio Oda, Isao Nakanishi (1994) Prognostic factors of colorectal cancer: K-ras mutation, overexpression of the p53 protein, and cell proliferative activity. Journal of Surgical Oncology;Volume 57 (1): pp 57–64.

57. J Gao1, Z Li, Z Chen, J Shao, L Zhang et al. (2006) Antisense Smo under the control of the PTCH1 promoter delivered by an adenoviral vector inhibits the growth of human pancreatic cancer. Gene Therapy 13, 1587–1594. DOI:10.1038/sj.gt.3302816.

58. Sarah P. Thayer, Marina Pasca di Magliano, Patrick W. Heiser, Corinne M. Nielsen, Drucilla J. Roberts et al. (2003) Hedgehog is an early and late mediator of pancreatic cancer tumorigenesis. Nature 425, 851-856, DOI:10.1038/nature02009.

59. Jeffrey Trent, Paul Meltzer, Mark Rosenblum, Griffith Harsh, Kenneth Kinzler et al.(1986) Evidence for rearrangement, amplification, and expression of c-myc in a human glioblastoma. Proc. Natl. Acad. Sci. USA, Vol. 83, pp. 470-473.

60. Georg Feldmann, Surajit Dhara, Volker Fendrich, Djahida Bedja, Robert Beaty (2007) Blockade of Hedgehog Signaling Inhibits Pancreatic Cancer Invasion and Metastases: A New Paradigm for Combination Therapy in Solid Cancers. Cancer Research, 67:2187-2196, DOI:10.1158/0008-5472.CAN-06-3281.

61. Erika Lindstro¨m,Takashi Shimokawa, Rune Toftga˚rd, Peter G. Zaphiropoulos (2006) PTCH Mutations: Distribution and Analyses. Human Mutation; Volume 27(3), 215 - 219.

62. Cheng-Jeng Tai, Chun-Chao Chang, Ming-Chung Jiang, Chung-Min Yeh, Tzu-Cheng Su et.al. (2012) Clinical-Pathological Correlation Of K-Ras Mutation and Erk Phosphorylation In Colorectal Cancer. Pol J Pathol; 2: 93-100.

63. Frédéric Varnat, Irene Siegl-Cachedenier, Monica Malerba, Pascal Gervaz, Ariel Ruiz i Altaba (2010) Loss of WNT-TCF addiction and enhancement of HH-GLI1 signalling define the metastatic transition of human colon carcinomas. EMBO Mol Med: 2(11): 440–457.
